# Laser Spectroscopy for Atmospheric and Environmental Sensing

**DOI:** 10.3390/s91210447

**Published:** 2009-12-22

**Authors:** Marc N. Fiddler, Israel Begashaw, Matthew A. Mickens, Michael S. Collingwood, Zerihun Assefa, Solomon Bililign

**Affiliations:** 1 NOAA-ISET Center, North Carolina A&T State University, 1601 E Market Street Greensboro, NC 27411, USA; E-Mail: mnfiddle@ncat.edu; 2 Department of Physics, North Carolina A&T State University, Greensboro, 1601 E Market Street, Marteena Hall, Greensboro, NC 27411, USA; E-Mail: israelncat@gmail.com; 3 Department of Chemistry, North Carolina A&T State University, 1601 E Market Street, New Science Building, Greensboro, NC 27411, USA; E-Mail: mamicken@ncat.edu; 4 Energy & Environmental Systems Program, North Carolina A&T State University, 1601 E Market Street, Greensboro, NC 27411, USA; E-Mail: mscollingwood@gmail.com

**Keywords:** LIF, LEAFS, LIBS, CRDS, photoluminescence, laser, environment, VOCs

## Abstract

Lasers and laser spectroscopic techniques have been extensively used in several applications since their advent, and the subject has been reviewed extensively in the last several decades. This review is focused on three areas of laser spectroscopic applications in atmospheric and environmental sensing; namely laser-induced fluorescence (LIF), cavity ring-down spectroscopy (CRDS), and photoluminescence (PL) techniques used in the detection of solids, liquids, aerosols, trace gases, and volatile organic compounds (VOCs).

## 1. Introduction

There are numerous traditional optical, gas chromatographic and mass spectrometric methods that have served extremely well in the detection of atmospheric and environmental trace gases, solid, and liquid compounds. However, promising new sensing and analytical measurement techniques based on laser spectroscopy have emerged and have been successfully used in numerous applications.

There has been an exponential growth in the application of laser spectroscopic techniques in almost every area of pure and applied science. This interest has spurred recent developments in several novel technologies, such as diode and fiber lasers for the optical communications industry, diode-pumped solid-state lasers, *etc.* These advances, coupled with the reduced cost and complexity of laser systems, make such spectroscopic sources more universally available and user friendly to both established and new fields. These fields include monitoring air and water quality; industrial, traffic, and rural emissions; atmospheric chemistry; chemical analysis and process control; medical applications and cancer recognition; applications to national security and explosives detection; vegetation remote sensing; artwork characterization, *etc.* This has also led to significant economic growth as the global market for environmental sensing and monitoring technologies was worth $9.1 billion in 2008 and an estimated $10.1 billion in 2009. This expected to reach $13 billion in 2014, for a compound annual growth rate (CAGR) of 5.2%.

It is evident that use of lasers and laser spectroscopic techniques in atmospheric and environmental sensing continues to grow. This review selectively covers some of the applications of these techniques including laser-induced fluorescence (LIF), cavity ring-down spectroscopy (CRDS) and photoluminescence techniques (PL). The section on CRDS covers the literature since 2005, while the sections on LIF and PL cover progress since 2000.

The first section of the review covers recent development in the area of LIF that have bearing on environmental analysis. The LIF technique is used widely in research for a variety of analytical applications, from interrogation of plasma plumes in Laser Induced Breakdown Spectroscopy (LIBS), to determination of cancerous tissues, to fluorescence spectroscopy of single molecules. LIF is one of the most sensitive approaches available for analytical purposes. The application of LIF techniques to the study of problems related to atmospheric and environmental sensing is reviewed.

The CRDS section covers a brief summary of some of the common experimental schemes that have been used in various studies. Covering experimental setups is essential for CRDS, since it is a relatively new technique (only about 20 years old) and its application is expanding. The rest of the section is devoted to discussing the atmospheric and environmental applications of CRDS-based techniques. The discussions will focus on trace gas detection or analysis, biologically relevant trace gas sensing, isotope ratio measurements, and aerosol studies.

The last section of this review is devoted to literature reports of PL complexes that exhibit “vapochromism/vapoluminescence” behavior and are relevant to chemical sensing. Emphasis is placed on the solid state complexes, and the molecular interactions with volatile organic compounds (VOCs) that permit analyte recognition through observed changes in PL properties. This section reviews the numerous investigations that examine the photophysical properties of fluorophores that can potentially be employed as efficient chemosensors. Since the PL process has now become a routine spectroscopic technique, no effort is made in this review to describe the technical aspect of the methodology.

## 2. Laser-induced Fluorescence Spectroscopy

### 2.1. Introduction

Laser-induced fluorescence spectroscopy is based on the electronic excitation of an atom or molecule by laser irradiation. When the electron returns to a lower-lying energy level, the energy may be released in the form of a photon. This forms the basic principle of fluorescence. The LIF technique is well established, and theoretical, mathematical, and practical treatments of LIF are available in several books and review articles [1-4]. Therefore, those subjects will not be enumerated here. This section of the review covers laser-based fluorescence techniques that have bearing on environmental analysis.

Many spectroscopic techniques are available for the analysis of a variety of systems, and the reader is directed to several reviews of other spectroscopic and spectrometric techniques not covered in this review [5-18].

Discussions of methods of quantitation, certified reference materials, sample preparation, and other topics are covered by a yearly review of atomic spectrometry techniques [5-9,16,17]. A recent review has been written concerning digestion procedures for soils, sediments, coal, and other materials [19].

This section focuses on the applications of LIF-based techniques, covering the literature since ∼2000. A brief functional description of LIF-based techniques is first given, with references for more in-depth treatments. The subsection on solids covers the *in situ* analysis of sediments and minerals, environmental transport of thallium, arsenic analysis and speciation, and characterization of plant tissue. The subsection of liquids analysis focuses on *in situ* analysis of fresh water and seawater, as well as the utilization of sample substrates. The subsection concerning aerosols and gases focuses largely on single-particle analysis of aerosols and the measurement of OH, HO_2_, RO, and RO_2_ radicals.

### 2.2. Review of the Technique

#### 2.2.1. Laser-induced Fluorescence (LIF) for Gas Phase Samples

Instruments for LIF are varied and frequently tailored to their application. All methods have several things in common: a laser source, a compartment for enclosing the sample, and a detector. Excitation/detection schemes are also varied and experiments may employ resonance, double resonance, two color, off resonance, and other means of excitation. Thought must be given to the sensitivity of the technique, but cost, size, and technical complexity also factor into design decisions. To achieve the lowest limit of detection (LOD), the excitation and detection frequencies that produce the most intense emission are typically chosen. However, this may not always be the best method. Spectral interferences can hamper detection at some emission lines and not others. In other instances, LOD is not an issue, and linear dynamic range or measurement accuracy is more desirable. This is why experimental setups vary, not only due to the substrate, but the overall goal of the technique.

#### 2.2.2. Laser-Excited Atomic Fluorescence Spectroscopy (LEAFS) for Solids

Laser excited atomic fluorescence spectrometry (LEAFS) is a technique in which volatilized atoms are characterized by the emission of radiation from an excited state that is induced by a laser source [20]. The distinction between LEAFS and atomic fluorescence spectrometry (AFS) is that the atomic excited states are produced from a coherent laser source with a narrow frequency profile, rather than incoherent, broad-band light source. Many papers use LEAFS and LIF interchangeably when referring to atomic systems, since LEAFS is a type of LIF. In this work, LEAFS will be used when referring to atomic species.

**Figure 1.**
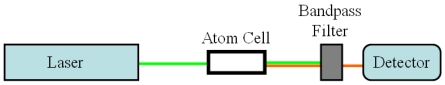
A general schematic of the experimental setup for LEAFS analysis.

The advantages of LEAFS stem from the high degree of sensitivity and selectivity resulting from the selection of excitation frequency and fluorescence frequency [21-23]. The extreme selectivity of the technique makes multi-element measurements cumbersome, though there are recent advances in “multidimensional” detectors [24]. Additionally, a degree of technical complexity is inherent in the system. A general schematic for LEAFS is depicted in Figure 1, where laser irradiation should be tunable between 180 and 800 nm [25]. The laser pulse passes through a cell that contains and/or produces gas phase atomic elements. The resulting fluorescence is carefully selected and detected. Unlike typical LIF experiments, laser intensity in LEAFS is typically set slightly above the limit of saturation in order to reduce the absorption of fluoresced radiation by unexcited species and to minimize the effects of fluctuations in laser intensity [23]. The deleterious effects of non-linear phenomena are minimized, while minimizing self-absorption produces a typical linear dynamic range of 5–7 orders of magnitude.

Unlike atomic emission spectrometry (AES) and AFS, atoms do not fluoresce unless they are excited by laser radiation. A comparison with other laser-based atomic spectroscopy techniques has been made by Sjöström [22]. Since this review is not focused on instrumentation or theory, readers should consult other papers on these subjects [20,21,23,26-29].

The elements investigated by LEAFS must be in the gas phase. If the sample has insufficient vapor pressure, energy must be imparted to the sample to volatilize the element. Volatilization techniques include simple effusive cells, flames [26,30-32], electrothermal atomizers (ETA) [21,33,34], glow discharge cells, inductively coupled plasma (ICP) [20,35,36], and laser ablated (LA) plumes. In general, the highest sensitivities are achieved using ETAs due to their small cavity volume, while plasmas are not as attractive due to the high intensity of background radiation [28]. Reviews on vapor generation of individual elements are covered elsewhere [15], as are various aspects of sample preparation.

The detection system usually consists of a narrow bandpass filter or monochromator, followed by a photomultiplier tube and boxcar integrator [20]. The resulting signal is than digitized and stored for further data analysis. Recent papers highlight statistical approaches in data analysis [37], LOD determinations [38-42], and multivariate analysis [43].

#### 2.2.3. Laser-induced Breakdown Spectroscopy (LIBS) for Solids, Liquids, and Aerosols

Another recent spectroscopic technique is laser-induced breakdown spectroscopy (LIBS), which is sometimes called laser-induced plasma spectrometry (LIPS) [44]. Though still in its infancy, LIBS is the direct descendant of LA-LEAFS. Whereas LA-LEAFS uses two lasers to accomplish ablation and spectroscopic characterization, it was realized that the wavelength of light was not of primary importance to the ablation process. This development leads to LIBS, where a single laser pulse forms a plasma, on a solid surface or within a gas or liquid, that results in volatilization and emission from atomic species. A recent review by Russo *et al.* discusses the fundamentals of the ablation process in LIBS [45]. Other recent reviews discuss the instrumentation and portability aspects of LIBS [46-48], detection systems [49], and fundamental principles and spatial resolution [13,15,24,50]. Other recent reviews on LIBS analysis of aerosols and gases focus on statistical methods for data processing [51-56], matrix effects on quantitation [48,57-59], and the effects of sample inhomogeneity on measurement uncertainty [60,61].

Another review compares LIBS and other laser techniques to the “super stars” of ETA-AAS, ICP-AES, and ICP-MS [62]. Several papers make a direct comparison of LIBS to LA-ICP-MS [63-65]. In general, the advantages of LIBS is its ability to detect substrates remotely (tens of meters for solids and meters for liquids) [66], its high spatial resolution, its lack of sample preparation, its potential portability [67], and its fast analysis time. In comparison to other elemental analysis techniques, however, its LOD is quite poor [15]. It has been determined that selective volatilization plays the largest role in the variability of quantitative LIBS measurements of solid materials [68]. Though instrumentation is not the focus of this work, the authors acknowledge recent advances in hybrid instruments that utilize both LIBS and Raman spectroscopy [69-71].

Due to the relative youth of the LIBS technique, most papers are instrumentation-based, which is not the focus of this review. Most applications concern the analysis of well-characterized surfaces, such as metal alloys. Analysis of glasses and ceramics, both modern and archaeological, are popular, since the ablated mass is so minimal that the method is considered non-destructive [10-12].

### 2.3. Applications

#### 2.3.1. Solids

Many applications of laser spectroscopic methods to the analysis of environmental solids are presented in Table 1. Of note are the recent papers evaluating the plausibility of *in situ* measurements using LIBS. Several papers focus on characterization of materials submerged beneath fresh or salt water. These include sediments [72], wood, and marble [73]. Ba, Mn, and Ti were detected, though calibration was hampered by shot-to-shot variability. The natural softness and roughness of the sample caused problems for laser focusing and plasma generation. Additionally, laser interrogation produced clouds of particles above the sediment surface, which scattered the laser and fluoresced light. Some detection thresholds were improved by signal processing [74]. *In situ* analysis of the seawater itself is discussed in the next section.

A series of recent publications from Cheam and coworkers involves measuring thallium (Tl) in sediments [94], water [95,96], and aquatic organisms [94,96,97] using LEAFS. Thallium has been scarcely studied due to its low concentrations and poor sensitivity in comparison to its more popular family members (Hg, Cd, and Pb), despite the fact that Tl possesses comparable toxicity [97]. Their investigations of coal power plants and mines suggest that coal type (rather than quantity) and/or geological contributions are responsible for the high Tl concentrations observed [95], though coal power plants and mines contain higher thallium concentrations than the other ecosystems studied [96]. Interestingly, by studying the Tl concentrations in aquatic organisms, they found that sites of maximum metal concentrations were not necessarily sites of maximum bioavailability and sediment concentrations are poor indicators of environmental distress [96,97].

A recent review by Simeonsson and collaborators discusses the use of laser spectroscopy techniques for arsenic analysis [98]. A number of atomization sources are discussed, including ICP, ETA, rhenium-coil, LA, along with hybrid techniques involving hydride generation (HG) and high performance liquid chromatography (HPLC). Limits of detection range from 4 (ICP) to 0.0003 ng/ml (HG) for aqueous samples [99-104]. Results were compared to other laser-based techniques and non-laser-based techniques, notably AFS and ICP mass spectrometry [98]. The majority of these studies, however, have been in aqueous solutions, though a few have studied diluted blood plasma [104-107] and plant tissue digests [98]. An LA-flame-LEAFS approach was applied to As on glass slides, which has yielded LODs of 0.02-0.5 μg/g ablated material [98, 102]. The uncertainty in this value is due to the uncertainty in the ablation spot diameter. Another recent review on arsenic speciation was conducted for the literature between 2000 and 2003 [108]. Others focus on the speciation of As^III^, As^V^ and other forms of arsenic in water samples, using a variety of spectroscopic techniques [13,109-111].

LIBS was recently used to determine Si/Ca and Ca/Mg ratios, in order to distinguish between sandstone, limestone, marble, and mortar [112]. The technique was tested on the façade of a cathedral, which identified the materials of the building with good resolution over a surface of 900 m^2^. The analysis of distant objects has been reviewed recently, focusing on experiments in which light was transmitted through the atmosphere, rather than an optical fiber [66]. The review of so-called “stand-off” LIBS discusses issues pertaining to remote measurement, including laser characteristics and optical systems associated with laser focusing and collection of fluoresced light. Applications of LIBS to non-flat [113] and moving [114] surfaces have recently been pursued. There is also a recent evaluation of common matrix effects encountered when performing LIBS analysis of geological materials [115].

Recent work has been done on the LIBS analysis of organic and biological materials [116]. An inherent shortcoming of this method is that these substances are largely composed of carbon, hydrogen, oxygen, and nitrogen. Relative signal intensities have been used to determine empirical formulas, though difficulty arises due to significant interferences from atmospheric nitrogen and oxygen. Emission from C_2_ and/or CN has allowed the differentiation between organic and inorganic samples.

LIBS has also made headway in the analysis of biological samples, though only papers that are environmentally relevant are presented here. One paper studies uptake of lead into *Helianthus annus* [117]. Plants were hydroponically grown in different concentrations of lead acetate and dried leaves were analyzed directly using LIBS. Pb uptake was shown to alter the spatial distribution of Mn and K in leaf tissues. Another study used the upper surfaces of leaves as substrates for accumulating atmospheric aerosol [118]. Aerosols that had undergone dry deposition on *Sophora japonica* leaves were evaluated for their metal content using femtosecond-LIBS.

#### 2.3.2. Liquids

There are several recent methods for the *in situ* analysis of water. In two laboratory studies, LIBS was used to detect dissolved Ca, K, Li, Mn, and Na [119,120]. Pressures of up to 272 atmospheres (4000 psi) were tested, with no pressure effect on Ca or Na. The emission of Mn actually increased with increasing pressure. Additional salinity (NaCl) increased the signal for Ca and had no effect on Mn or K. A paper investigating the matrix effects in LIBS for the analysis of potassium in water droplets has a heavy bearing on seawater analysis [121]. Several other studies on water and seawater are presented in Table 2.

A promising method has recently been employed that uses wood substrates for water analysis [131]. With water absorbed into the wood, LIBS was used to quantify heavy metals such as Cr, Mn, Cu, Cd, and Pb. LOD were between 0.029 and 0.59 mg/L, which is 2–3 orders of magnitude more sensitive than LIBS without a substrate. Other recent publications utilize a substrate to provide a restricted space for the sample, such as a metal plate with 100 μm holes for the analysis of soils by LIBS [132]. The authors claim that this system reduces lost sample particles during irradiation and that atomization and excitation are enhanced due to sample confinement in the surrounding metal.

#### 2.3.3. Aerosols and Gases

Laser-based fluorescence techniques have recently been applied to aerosol analysis. Much of this work has been spurred by tighter regulation of point sources of metals, such as furnaces and incinerators. The majority of studies concerning the bulk analysis of aerosol use X-ray fluorescence or ICP-MS, while single-particle analysis is dominated by aerosol mass spectrometry. However, the ability to perform (near) real-time analysis in the field has made LIBS and LEAFS attractive techniques. Several examples of aerosol analysis are shown in Table 3. LIBS analysis of biological aerosols (spores, pollen, *etc.*) suffers from the same problems as other organic and biological samples (discussed above). For this reason, research on aerosol analysis is gravitating to other techniques or LIBS in parallel with other techniques [133].

Recent advances in utilizing Raman microscopy and electron microprobe analysis (EMPA) have allowed characterization of single aerosol particles [138]. The combined techniques yield information on size, morphology, elemental and molecular composition, and molecular structure for particles as small is 500 nm in diameter. Particles were transferred from an impactor to a thin glass needle and piezo-driven nano-manipulators allowed sequential analysis of single aerosol particles by both techniques. Much of the recent literature has not focused on novel applications of LIBS to aerosols, but on technical aspects of analysis.

Laser-based spectroscopic techniques have also been applied to the analysis of trace gases in the atmosphere. Several recent reviews discuss techniques for the analysis of HO_x_ (OH^•^ and HO_2_^•^ radicals) [139,140]. Shortly after recognizing the importance of OH^•^ in the atmosphere, LIF measurements of OH^•^ were performed in 1972 [141]. The technique has since matured significantly. LIF measurements are typically performed in reduced pressure chambers and the method is also known as fluorescence assay by gas expansion (FAGE). Specific experimental setups for LIF OH^•^ measurements are numerous, but all modern instruments possess a gas expansion region, a high repetition rate laser system, an electronically-gated detector (photomultiplier or microchannel plate) that is operated in photon counting mode, and a calibration system [140]. Since the lifetime of OH^•^ is ∼1 second, LIF offers the advantage of *in situ* measurement, fast analysis time, and excellent sensitivity. Selectivity is also very good. Fluorescence from other species at 308 nm, such as SO_2_ and formaldehyde, are discriminated against by delayed gating. The only major interference is O_3_. LIF instruments are, however, costly and have sufficient size and weight to make field measurements cumbersome (*i.e.*, the instrument is usually housed within a small flat-bed trailer with a second trailer for the roots pump). The largest disadvantage of the technique is its need for calibration, as measurement accuracy is typically limited by the certainty of the OH^•^ calibration source [140]. Typical LOD for OH^•^ measurement during the daytime is 1.4 × 10^5^ molecules/cc for S/N of 1 and a collection time of 300 seconds [139,140]. Smaller LOD are achievable at night.

Another laser spectroscopic technique is also commonly used for OH^•^ measurement: long-path differential optical laser absorption spectroscopy (DOAS). Though DOAS is not covered in this review, a recent comparison between DOAS and LIF has been made [142]. LIF was found to have greater precision and time resolution, while DOAS is regarded as a primary standard due to its greater accuracy. Excellent agreement was found between the techniques, though LIF measurements were somewhat higher under high NO_x_ conditions. Numerous calibration instrument have been devised, including those that involve photolysis of water, measurement of hydrocarbon decay rates, and generation of OH^•^ through ozonolysis of alkenes [140,143,144]. Typically, several of the aforementioned methods are used. A new calibration source for HO_2_^•^ has been devised, which uses UV photolysis of water over a TiO_2_ catalyst [145]. Instrument intercomparisons have been made for ground [140], laboratory [144], and aircraft-based instruments [140]. Measurement of HO_2_^•^ and RO_2_^•^ radicals typically involve chemical conversion to OH^•^ followed by LIF detection [146,147] and new developments in this area are continuing [148].

Several recent field experiments and campaigns utilizing LIF for OH^•^ measurement are referenced here [149-152]. Since findings from these campaigns are typically lengthy, involve numerous measurements on a variety of compounds, and are somewhat specific to the regions they study, findings from these works will not be enumerated here.

## 3. Atmospheric Sensing based on Cavity Ring-Down Spectroscopy

### 3.1. Introduction

Cavity ring-down spectroscopy is an ultra sensitive technique that is currently being used in various disciplines and environments. Since its introduction by O'Keefe and Deacon in 1988 [153], it has seen a growing number of applications and variations. There are now several portable instruments that use the technique, or some modification of it, for a variety of purposes. This section of the article will first briefly summarize some of the common CRDS experimental schemes that have been used in various studies. The rest of the section will be devoted to discussing the atmospheric and environmental applications of CRDS-based techniques. The discussions will focus on trace gas detection or analysis, biologically relevant environmental sensing or breath analysis, isotope ratio measurements, and aerosol studies. Due to the remarkable growth of the technique and the resulting large volume of literature available on different applications and techniques of CRDS, this review will be neither thorough nor exhaustive. However, the reader will be provided with a large number of references for further investigation.

### 3.2. General Principles and Techniques

In principle CRDS is a direct absorption technique in which the change in the intensity of light passing through a sample of interest is measured. Most direct absorption techniques involve measuring the incident and transmitted intensity of light passing through a sample. Therefore, the sensitivity of such techniques is limited by the minimum detectable change in intensity. In addition, direct absorption techniques are not zero background techniques and, as a result, have limited capability in detecting small changes in the light intensity as it goes through the sample. Multi-pass configurations, such as White or Herriot cells, are usually employed to improve the sensitivity of direct absorption techniques. CRDS chartered a new course in absorption spectroscopy by measuring not the change in intensity of light, but the rate of decay of the intensity of light leaking out of a high-finesse optical cavity. The rate of decay is a function of the cavity length, cavity transmission, and the absorptivity of the sample. If measurement is taken without the absorber being present and a second measurement with the absorber present in the cavity, the difference in the rate of decay will be solely due the absorber. From this, spectral information or concentration of the absorber can easily be determined.

The growth of CRDS use is a remarkable one. Its wide ranging application is also noteworthy. There are several superb review articles that explain the technique and the theoretical underpinnings [154-156]. Although early measurements were done on gas phase systems, there are now several studies that have successfully applied the technique to condensed phase and solid films systems. The history and development of the technique is well documented by Scherer *et al.* [155] and more recently by Paldus and Kachanov [157]. There are also application and technique-specific reviews available in the literature. For example, Brown [158] and Atkinson [159] review the application of CRDS-based methods to atmospheric and environmental studies. Ball *et al.* [160] give a detailed analysis of CRDS using broad-band light sources. Mazurenka *et al.* [161] give detailed analysis of CRDS and cavity enhanced spectroscopy, a variation of CRDS, using diode lasers.

While pulsed laser sources were used in early works using CRDS [153] several variations have since been developed. Some of these developments include Broad-Band CRDS (BB-CRDS) [160,162-164], Continuous-Wave (CW-CRDS) [165-168], Cavity-Enhanced Absorption Spectroscopy (CEAS) [161,169] (sometimes called Integrated Cavity Output Spectroscopy (ICOS)) [170,171], and Phase Shift CRDS (PS-CRDS) [163,172,173]. In addition the use of CRDS in liquids and thin films has led to the development of Evanescent Wave CRDS (EW-CRDS) [174]. In most CRDS applications the use of lasers, CW or pulsed, is very typical. However incoherent and coherent non-laser sources of light have been successfully applied in CRDS-based techniques for a variety of applications. Light-emitting diodes (LED) have been used for trace gas analysis [175-179] using a BB-CEAS methodology. The use of supercontinuum radiation sources is also a recent advancement in cavity-based applications [180-183].

Some of the major advantages of CRDS include its experimental simplicity, especially for pulsed CRDS, its high sensitivity due to the multi-pass nature of the optical cavity, and its independence from laser intensity fluctuations. Light travels back and forth in the optical cavity and results in a large effective path length. The technique can be used to investigate gaseous, liquid, and thin filmed solid species with no or little sample preparation. The advances in lasers and laser technology have allowed for fairly rugged portable instruments that can be used in field measurements. With appropriate data acquisition and signal processing, CRDS systems are capable of furnishing results in real time. On the other hand, unavailability of lasers in all spectral regions is a limiting factor for CRDS applications. In addition, the highly reflective mirrors maintain their reflectivity only over a small wavelength range, making the spectral measurements of more than one species difficult. The cost associated with laser sources and highly reflective mirrors presents a barrier to its application.

### 3.3. Sensing Applications of CRDS

As mentioned above, CRDS and its variants have been successfully applied in several disciplines. The following sections attempt to summarize recent studies that have been carried out using the CRDS technique in atmospheric and environmental applications. Additionally, applications for biological sensing of atmospherically related compounds will also be reviewed.

#### 3.3.1. Atmospheric Sensing

The CRDS technique has, perhaps, found its most extensive use in atmospheric studies. The past decade has seen an exponential increase in the number of studies that use the technique. The advent of technology and the innovations of schemes that result in experimental stability, simplicity, and portability have increased its applicability for scientists and industry professionals. Its high sensitivity allows for measurement of the very weak absorptions and very dilute concentrations that are typical for trace gases. In addition, the technique's sensitivity has been used to probe the photochemistry of the atmosphere and has provided valuable detail to our understanding of atmospheric chemistry. Although there are diverse atmospheric applications of the technique, this review will focus on mainly trace gas detection, aerosol characterization, and isotope ratio measurements. Other atmosphere applications include atmospherically important chemical reaction and kinetics studies [184-186] and studies of photochemical properties of atmospherically relevant molecules [187-190]. There have been multiple reviews of the application of CRDS to atmospheric and environmental studies [158,159]. In fact this review is structured similar to that of Brown [158]; however, deliberate effort is made to focus only on the studies that have been conducted since 2005.

##### 3.3.1.1. Trace Gas Detection

CRDS is one of the spectroscopic techniques for the study of trace gases. Of course, trace gases are gaseous substances which are less than one percent by volume of the atmosphere of the earth. Since the beginning of industrialization, there has been an increase in many trace gases due to anthropogenic sources. These gases include ozone, sulfur dioxide, nitrogen oxides, and methane; among others. Different types of CRDS variations have been used to probe a host of trace gases and there are now commercially available CRDS instruments for their measurement [191-193].

###### 3.3.1.1.1. Water

Water vapor absorption has been extensively studied for quite a number of years. However, the paramount significance water has to life requires that we continue to study and perfect our understanding of water cycling. Although Fourier Transform Spectroscopy (FTS) has been the traditional method of studying water, CRDS has provided an alternative. Dupre *et al.* [194] reported a study on the spectrum of water in the blue region. Their CW-CRDS setup used an Ar^+^ laser pumping a Ti:sapphire laser, which is frequency doubled using an external intracavity LBO crystal. They were able to see 62 lines with a sensitivity of approximately 1 ppb. High resolution spectra of water were measured using a frequency-stabilized CRDS that used a continuous wave external cavity diode laser (CW-ECDL). The high resolution study demonstrated the capability for detecting subtle changes in the line shape and line position of a spectrum [195].

Crosson of Picarro [196], one of the companies that now manufactures ready-to-use CRDS-based trace gas sensors, recently reported on the field trials of their instruments conducted by Pennsylvania State University and the National Oceanographic and Atmospheric Administration (NOAA). The instrument uses two tunable distributed-feedback diode lasers and a three mirror cavity setup (i.e. with mirrors in an isosceles triangle configuration), and is able to measure simultaneously and *in situ* CO_2_, CH_4_, and H_2_O. The instrument's typical sensitivity was reported to be 1.6 × 10^–11^ cm^–1^·Hz^–1/2^ and possessed detection limits of 50 ppm for H_2_O.

####### 3.2.1.1.2. Carbon Dioxide, Methane, and Carbon Monoxide

The Picarro instrument mentioned above also measured CO_2_ and CH_4_ with detection limits of 100 and 0.5 ppb, respectively. The instrument showed good agreement with traditional methods of detection (non-dispersive infrared spectroscopy), with the benefits of the CRDS instrument requiring less frequent calibrations. The application of extended-wavelength distributed feedback (DFB) diode lasers in CEAS was first reported by Kassi *et al.* [197]. They used diode lasers in an optical feedback CEAS (OF-CEAS) setup to undertake *in situ* measurements of geothermal gases from a volcano. They specifically monitored methane and carbon monoxide concentrations. The DFB diode lasers emitting at 2.33 μm achieved a sensitivity of 3 ppm for CO and 75 ppm for CH_4_. Traditionally, volcano gases were monitored through either gas chromatography (GC) or mass spectrometry (MS) which required sample collection in the field and analysis in the lab. The CEAS instrument, weighing 16 kg and fitting inside a 48 cm chassis, allows for *in situ* measurements to be made in the field.

A minimum detectable concentration of 850 pptv (parts per trillion by volume)·Hz^–1/2^ for methane was reported by Malara *et al.* [198] using an off-axis integrated-cavity-output spectroscopy (OA-ICOS) scheme. A diode laser amplified by an external Yb-doped amplifier operating in the interval 1,545–1,605 nm was scanned over the molecular transition. They demonstrated the potential for a similar setup to detect a number of species including C_2_H_4_, NH_3_, and N_2_O. A recent study by Orphal and Ruth [199] reports a new spectroscopic mechanism where they combine FTS with CEAS using an incoherent light source (Xe arc lamp). The performance of the technique was demonstrated by measuring the overtone bands of CO_2_, OCS, and HD^18^O between 5800 and 7000 cm^–1^.

Quantum cascade lasers (QCL) provide new possibilities for highly selective trace gas detection in the mid-infrared (MIR). Thermoelectrically (TE) cooled pulsed and CW-QCLs were used by Welzel *et al.* [200] to demonstrate potential applications. Over a 20 s integration time, methane was measured at 7.42 μm for a detection limit of 6 × 10^8^ molecules•cm^–3^ and N_2_O was measured at 8.35 μm for a detection limit of 2 × 10^9^ molecules•cm^–3^. Residual mode noise of the cavity limited them from achieving lower limits of detection. In addition, the TE cooled pulsed QCLs suffered from intrinsic frequency chirp, which resulted in a need for frequent calibration.

A recent study [201] used CRDS to measure four combustion gases (CO, CO_2_, HCN, and C_2_H_2_) for remote fire detection. The portable CRDS system uses two fiber-coupled near-infrared distributed feedback lasers. One operates at 6361 cm^–1^ and is used to detect CO and CO_2_ with a tuning range of 16 cm^-1^. The instrument was tested along with traditional fire detection mechanisms. While the CRDS system provided rapid and simultaneous measurements of the combustion products, its sensitivity was not comparable to other instruments. However, the system was able to yield information on the nature and fuel of the fire, and also the presence of toxic materials. Being able to monitor multiple combustion gases also allowed for better decision making in regards to false alarms that can be initiated from monitoring only one indicator.

Other hydrocarbons have been studied using CRDS instruments. Pradhan *et al.* [202] have successfully demonstrated the use of a DFB diode laser in CW-CRDS setup to achieve limits of detection of 35 ppt for acetylene, after sample pre-concentration. By using vibrationally-damped optical mounts and a new detector, the authors were able to achieve an improved limit of detection of 8 ppt [203]. The new instrument was also fully automated, including the sample pre-concentration. A CEAS has been used to study the absorption spectrum of formaldehyde in high resolution [204]. The spectrometer uses two laser sources: a diode pumped Nd:YVO_4_ laser at 532 nm and an external cavity diode laser at 841 nm. The two laser sources were used in a sum frequency generation setup to produce narrow band radiation near 325 nm. A detection limit of 172 ppb for formaldehyde in atmospheric air was reported for the instrument.

####### 3.2.1.1.3. Oxides of Nitrogen

G. Schuster *et al.* [205] have used a novel method to improve typical CRDS and CEAS instruments for the measurement of ambient NO_3_/N_2_O_5_ outside of the laboratory setting. It entails heating the NO_3_ or N_2_O_5_ at the inlet so that thermal dissociation of the compounds can take place. The design is more rugged and inexpensive than typical instruments and very low concentration measurements are now possible with the technique. The only drawback is that only one of the gases could be measured at a time. In the future, the group expects to be able to measure both species simultaneously. They used a red-emitting laser diode as the light source with a center line at ∼662 nm. The investigators were able to detect concentrations as small as 2 pptv in ambient air [206].

There is yet another innovative improvement on CRDS technology that has allowed for the sensing of the NO_2_ trace gas. It was presented by J. Sato *et al.* and is based on CRDS using an optical fiber-coupled high-finesse cavity. It uses the spatial mode matching condition of the core of an optical fiber and a high-finesse external cavity. This setup allows for effective optical feedback into an antireflection-coated laser diode for stable resonant enhancement of the external cavity. The external cavity, which works as a ring-down cavity, could be remotely located from the light source and receiver section and connected by only a single mode optical fiber. The sensitivity was found to be 1.0 × 10^–7^ cm^–1^ in a compact 1-cm^3^ ring-down cavity volume [206].

A new design in CEAS has been put forward; it is termed supercontinuum CEAS (SC-CEAS). The primary advantage for using supercontinuum radiation sources is their broad wavelength coverage. Langridge *et al.* [181] used such an instrument to make quantitative measurements of NO_2_ and NO_3_. They were able to detect NO_3_ to a limit of 3 pptv in a two second integration time with a corresponding effective sensitivity of 2.4 × 10^–9^ cm^–1^·Hz^–1/2^ at 3σ noise level. Although the light source used (SC fiber laser) had an extended spectrum from 400 nm to 2,000 nm, the experimental investigation was limited by the available cavity ring-down mirrors to about a 100 nm spectral window.

A CEAS technique has been developed which uses a LED as a light source and the technique has been termed Incoherent Broad-Band Cavity Enhanced Absorption Spectroscopy (IBBCEAS). One study used a blue LED to project the light into the cavity [178]. The method has proven itself in the laboratory as it detects NO_2_ in the ambient air with a sensitivity of 18.1 ppbv for a 40 nm band width centered at 470 nm using moderately reflective mirrors (∼99.55%). Similar work was also done with NO_3_ [207]. Another group of researchers [208] used the IBBCEAS method to investigate NO_3_ and NO_2_ radicals *in situ* in the spectral range between 630 and 690 nm. Their 20 m long optical cavity instrument participated in a two week campaign for intercomparison of instruments for NO_3_ radical detection. Detection limits reported include 2 pptv for NO_3_ and 2 ppbv for NO_2_ with a 5 s acquisition time.

In their paper in Environmental Science and Technology, Gherman *et al.* [179] presented the application of IBBCEAS in the near-ultraviolet for simultaneous detection HONO and NO_2_. The technique developed by the same group uses incoherent broad-band light sources (such as LEDs) in a CEAS setup. The UV study used a high power LED and measured both HONO and NO_2_ between 360 and 380 nm. The reported limits for detection were 4 ppbv for HONO and 14 ppbv for NO_2_ with an integration time of 20 s.

####### 3.2.1.1.4. HO_x_ Radicals

HO_x_ radicals (OH +HO_2_) play a very crucial role in the chemistry of the atmosphere, since they are responsible for a great part of the atmosphere's oxidizing power. The study of these radicals has been the topic of many investigations. However, the short life time of OH (on the order of seconds) and its high reactivity has made measuring its concentration a challenge. CRDS-based techniques have demonstrated high sensitivity and rapid time response for detection of trace constituents and they have been applied for spectroscopic studies of HO_x_ radicals.

Thiébaud *et al.* [186] combine laser photolysis with near-infrared (NIR) CW-CRDS for spectroscopic and kinetic study of the HO_2_ radical [186]. A NIR distributed feedback diode laser was used for detection of the radicals formed in the laser photolysis stage. They were able to use different timing strategies to obtain kinetic information which they report are more efficient than previous setups. A CRDS setup has also been used to detect the HO_2_ radical in dielectric-barrier discharge plasmas [209]. This setup is also CW-CRDS and uses diode lasers that are however not DFB. The observation was made for mixtures of HCHO/O_2_/H_2_O/N_2_. The authors studied the temporal evolution of the radical and also the effects of the concentration of oxygen and water on the radical concentration. A very recent study [210] demonstrates the CW-CRDS measurement of the absolute concentration of OH radicals in an atmospheric pressure AC discharge.

##### 3.3.1.2. Aerosol Measurement

Atmospheric aerosols absorb and scatter solar radiation, thereby having a substantial influence on the radiative balance of the Earth's atmosphere. Aerosols also have an obvious effect on atmospheric visibility. A very thorough recent review article [211] details the study of aerosol light absorption. The optical properties of atmospheric aerosols are characterized by their extinction cross-section *σ_ext_*, which is the sum of the scattering *σ_sca_* and absorption *σ_abs_* cross section, and by their single scattering albedo (SSA) *ω*, which is the scattering to extinction cross ratio.

In the earliest application of CRDS for aerosol study [212], the extinction cross section was measured. The result of the study exhibited the potential applicability of the technique, but the sensitivities achieved were much lower than conventional particle-sizing instruments that operated by detection of particulate scattering from intracavity CW laser radiation. The application of CRDS was found to be more advantageous than laser-induced incandescence for measurement of soot volume-fractions [213]. Other aerosol studies using pulsed CRDS include [214-216]. In 2003, Strawa *et al.* reported [217] the first use of two CW laser sources (laser diodes) that could measure both the extinction and scattering coefficients, cross sections divided by volume. The portable instrument was tested in the laboratory with lab-generated aerosols and in the field with ambient air. The results from the study were in good agreement with the then state-of-the-art nephelometer. The airborne deployment of the improved instrument named Cadenza in the Atmospheric Radiation Measurements Aerosol Intensive Operating Period (ARM:AIOP) is documented by [218] and the comparison with other techniques is reported in [219].

Comparable accuracy was reported by Pettersson *et al.* [220], using a pulsed CRDS scheme along with a particle generation, sizing, and counting mechanism. Their system gives an absolute measurement of extinction with uncertainty determined mainly by statistical fluctuations in the number of particles in the laser beam. The system served as a basis for the field deployed instrument described by Baynard *et al.* [221]. The instrument makes simultaneous measurements at 355, 532, 683, and 1064 nm using an Nd:YAG laser with a Raman shifter.

In the Reno Aerosol Optics Study (RAOS) [222] three cavity ring-down instruments were used with several other techniques for comparative studies. One of the instruments used is the Strawa *et al.* instrument mentioned above [217]. The other two instruments are pulsed CRDS instruments, one of which had been reported earlier by Smith and Atkinson in 2001 [223]. However all three instruments recorded lower values for the aerosol extinction coefficients when compared with the sum of nephelometer scattering and photoacoustic absorption results which were used as references.

An instrument that employs a hybrid of CRDS and CEAS to measure aerosol extinction was presented by Moosmüller *et al.* in 2005 [224]. Another novel method that combines cavity ring-down with fluorescence spectroscopy was presented by Richman *et al.* [225] to measure absorption, scattering and fluorescence of aerosols using a CW laser source. The geometric design of the optical cavity afforded only part of the fluorescence to be collected from the optical beam path within the ring-down cavity which affected the ring-down time. The work shows the potential application for studying both the optical and chemical properties biological aerosols.

The use of CRDS for determining complex refractive index of pure and mixed aerosols, in addition to extinction cross section, was presented by A. Abo Riziq *et al.* [226]. The pulsed CRDS system's performance was tested with polystyrene spheres (PSS), ammonium sulphate (AS, (NH_4_)_2_SO_4_), sodium chloride (NaCl), glutaric acid (GA), and Rhodamine-590. The results reported were in good agreement with theoretical calculations and previously reported results. In a later study, Spindler *et al.* [227] combined CRDS aerosol optical extinction measurement with size distribution measurement to retrieve the complex refractive indexes. They tested their technique with AS and the water soluble dye nigrosin. They demonstrate that there is no need to map the extinction function of the aerosol over a large range of particles sizes, rather measurement of two size distributions and the respective extinction coefficients suffice to calculate the refractive index. Very recently, an improvement to the system by using a continuous wave laser source was reported [228]. Based on the several aerosol species they studied, the authors claim an almost two orders of magnitude higher sensitivity and four times faster repetition rate over the only other CW-CRDS system developed by Strawa *et al.* [217].

Butler *et al.* [229] use a recently developed variant of CRDS, optical-feedback cavity ring-down spectroscopy (OF-CRDS) to study extinction due to a single micron-sized aerosol particle. The CW laser source was a distributed feedback diode laser (DFB-diode laser) operating at 1,650 nm. The water aerosols were created using a compressor nebulizer and were flown across the laser beam perpendicular to its axis. The study shows that the extinction of the particles depends on their radial positions within the beam. In a later study [230] the authors attempted to use the technique to measure the extinction cross section caused by multiple, nearly monodisperse aerosol particles without the requirement of knowledge of the number density of the particles in the sample. However, the statistical model they presented significantly underestimated the extinction cross sections.

##### 3.3.1.3. Isotope Sensing

Isotope ratio measurement can furnish valuable information about the composition and also the chemical processes of the atmosphere. It is also a critical technique in bio-medicine. The variation in natural isotope ratios can help identify and quantify sources and sinks of different molecules [231]. The conventional method of isotope ratio measurement is the aptly named Isotope Ratio Mass Spectrometry (IRMS). However there are now instruments that offer comparable sensitivity and accuracy that are based on the cavity ring-down technique. Table 4 lists the different studies carried out for isotope ratio measurements.

Previously, studies done to identify isotopes using CRDS have proven to be inferior to mass spectroscopic methods [158]. However, reasonable results have since been obtained from the technique. The sample quantity required to detect isotopes using CRDS is significantly less than what is required for MS. Recent improvements to CRDS have allowed it to surpass the detection limits of MS techniques. Using a method known as wavelength-scanned CRDS (WC-CRDS, a group of designers and researchers have been able to distinguish between the four most stable isotopes in water; namely, deuterium, hydrogen, oxygen-16, and oxygen-18 [238].

There has been an improvement in the typical CRDS instrumentation where a piezodrive is attached the cavity mirrors so that better stabilization could be achieved. The piezodrive is basically an electro-mechanical devise which could be adjusted to improve the stability of the system. It is anticipated that this improvement will make CRDS determination of δ^13^C values comparable to or better than that of the best routine IRMS. It should also be noted that isotopic species that have nearly the same mass, such as ^13^C^16^O^17^O and ^12^C^16^O^18^O, are difficult to distinguish using IRMS techniques, whereas they are readily spectroscopically distinguishable using CRDS [232].

The researchers who used the WS-CRDS instrument claim that the performance of the WS-CRDS isotopic water analyzer matches or exceeds IRMS measurements in its ease of use and that it is less costly. WS-CRDS has a high precision when it has been used to analyze liquid samples with small quantities of deuterium (δD or δ^2^H) compared to a standard; in this case the researchers have measured δD at values less than 0.005; while, for oxygen-18 (δ^18^O), it was 0.001. When measuring ambient vapor samples they were each less than 0.01 and 0.002 for δD and δ^18^O, respectively. The WS-CRDS system is designed with an evaporator that homogenizes vaporized liquid water samples before they are tested by the gas-phase instrument. This allows measurement of the isotopic content of water, whether it is in liquid or vapor form. The evaporator enables automated switching between ambient atmospheric gas sampling and liquid samples. These measurements require no extensive sample preparation like MS does. Researchers have claimed that WS-CRDS has decisive advantages over the traditional approaches. It is also beginning to prove itself as a high precision method by which reliable and repeatable detection and analysis are possible. There are some limitations with the method, though. Future improvements should include investigation and modifications to reduce memory effects within the WS-CRDS instrument as well as the evaporator. A reduction in sample memory would reduce the sampling time and hence increase the throughput of the analyzer. The analyzer has proved reliable in both very humid and very dry field settings. The performance of the instrument deployed for *in situ* measurements in the field as well as in a laboratory setting has exceeded expectations.

#### 3.3.2. Sensing of Atmospherically-relevant Compounds from Biological Sources

An exciting area of development for spectroscopic techniques based on a high-finesse optical cavity is in their biological/medical applications. Each application discussed in this subsection (CO_2_ stable isotope analysis, nitric oxide and hydrocarbon measurement), is also relevant toward atmospheric trace gas studies.

Modern medicine operates on the principle that detection of different biomarkers can yield information for diagnostic purposes. One of these detection practices is through breath analysis. Breath-analysis diagnostic methods do not enjoy the same level of use as other diagnostic methods (blood test, urine test, *etc.*) [239]. However, the advantages for breath analysis over other techniques are multifold. Breath analysis is non-invasive and inherently safe because the subject is only required to breathe. In this section we review the application of the CRDS technique for biological sensing, mainly for human breath diagnostic methods.

Early suggestion for the potential use of CRDS for human breath diagnostics were made by Kleine *et al.* [240]. In their study they measured ^13^CH_4_ in ambient air in real-time using cavity leak-out absorption spectroscopy (CALOS) with a tunable CW CO laser combined with a tunable microwave side-band generator. They were able to achieve detection limits in the hundred ppt ranges. A mid-infrared quantum-cascade distributed-feedback laser (DFB-QCL) used in a CRDS setup by Paldus *et al.* [241] showed that a detection limit of 0.25 ppb was possible for ammonia in nitrogen at standard temperature and pressure. The first application of detection using CRDS in actual exhaled breath sample was done Menzel *et al.* [242]. Studies on different molecules are discussed below.

##### 3.3.2.1. CO_2_ Stable Isotope

The determination of δ^13^C in human breath has the potential to serve as an indicator for some diseases. This is especially true for the *H. pylori* bacterium which is a leading cause of ulcers [243]. The presence of the bacteria can be determined from the difference over baseline (DOB) value for the δ^13^C in human breath. Crosson *et al.* [232] measured the δ^13^C values for subjects known to have the bacteria and those that did not. The instrument used an external cavity laser diode (ECLD) as a light source for a CW-CRDS setup. The reported minimum detectable absorption loss for the instrument was 3.2 × 10^–11^ cm^–1^·Hz^–1/2^. They succeeded in being able to identify subjects' breath that tested positive for the bacteria from the measured δ^13^C value for CO_2_. The CW-CRDS instrument was a less costly and more compact alternative to the traditional technique of IRMS, while providing comparable sensitivity. In 2006, Kasyutich *et al.* [233] presented their proof-of-concept study where they used an off-axis cavity-enhanced absorption spectroscopy (OA-CEAS). The distributed-feedback laser diode (DFB-LD) was operated near 1605 nm. However the sensitivity of their instrument was not sufficient enough to provide the precision needed for *in situ* measurement of δ^13^C. A recent study by Thrope *et al.* [244] used a novel technique which they call cavity-enhanced optical-frequency comb spectroscopy to measure a host of gases and also stable isotopes of CO_2_ across a 200 nm spectral window from human breath samples. Their demonstrative study shows the instrument has a sensitivity of 8 × 10^–10^ cm^–1^.

##### 3.3.2.2. Nitric Oxide

Nitric oxide (NO) is produced in the human body and controls different physiological processes. The detection of NO in exhaled human breath has been studied as an indicator for asthma and other respiratory inflammatory diseases [242]. NO is one of the well-studied biomarkers by cavity ring-down type techniques [242,245-250]. With the exception of two, all of the above studies came out of Rice University. Conventionally, NO in human breath is detected using chemiluminescence, however spectroscopic sensors of NO based on high-finess optical cavities are emerging as an alternative.

The first study of exhaled NO was conducted by Menzel *et al.* [242]. They compared the performance of two spectroscopy schemes: a 100 m optical path length multi-pass cell and a cavity-enhanced absorption spectrometer. They used a continuous wave quantum-cascade distributed-feedback laser operating at 5.2 μm. The sensitivities they reported (16 ppb for CEAS) fell short of the typical 1 ppb level achieved by chemiluminescence-based devices. However, they were clearly able to demonstrate the potential use of the technique for NO detection in human breath. In a later study, Kosterev *et al.* [245] were able to modify the scheme by manipulating the QC laser current for frequency tuning and laser emission interruptions. The attempt was made to detect NO at sub-ppm levels in pure dry N_2_, and in exhaled air samples where interference from H_2_O and CO_2_ needed to be considered. The obtained sensitivity of 0.7 ppb was very promising, however they were not able to detect NO in exhaled breath due to interference from H_2_O and CO_2_. They suggested that a WC laser capable of targeting a spectral region that does not suffer from H_2_O and CO_2_ interference can potentially detect NO down to sub-ppb levels.

The next development that came was the use an off-axis integrated-cavity output spectroscopy (OA-ICOS) scheme with a CW mid-IR DFB-QC laser [246]. This scheme was also combined with wavelength modulation spectroscopy and tested with nasal-exhaled air. The reported improvements over the CRDS setup include easier alignment and robust performance over a long time period. The detection noise equivalent sensitivity for the instrument was 10 ppbv. When combined with wavelength modulation, the sensitivity increased to 2 ppbv. A similar setup that uses a DFB-QC continuous wave laser operating at 5.47 μm was presented by McCurdy *et al.* [248], with a reported sensitivity of 3.6 ppbv at 3σ. This instrument was compared with a conventional instrument, based on chemiluminescence, used for detection of NO in human breath and was found to agree with a mean difference of only 0.6 ppbv. A later study [249] by the same authors compared the performance of a similar instrument using a QC laser at 5.22 μm for detecting both NO and CO_2_ in a single breath cycle. The comparison of results with those conventional detection instruments was found to be in good agreement.

Other studies on NO include the measurement of ^14^NO and ^15^NO in a CALOS setup using a CO laser [247]. This study reported a much improved detection sensitivity of 800 ppt for ^14^NO and 40 ppt for ^15^NO. Heinrich *et al.* [250] very recently reported the application of the improved instrument for simultaneous analysis of ^14^NO and ^15^NO in human breath samples. The noise equivalent detection limit was 6.6 ppt, the highest sensitivity reported so far.

##### 3.3.2.3. Ethane

Ethane in exhaled human breath has been studied as a useful marker of lipid peroxidation in the human body. The conventional method of detecting ethane in human breath is gas chromatography (GC) where the breath sample must be pre-concentrated. Typical ethane levels in human breath are in the ppb level and cavity ring-down spectroscopic techniques can be used as an optical ethane detection mechanism that does not require sample pre-concentration and can furnish results in real time [251].

The first of such an instrument using a cavity leak out spectroscopy setup was reported in 2002 by Popp *et al.* [252]. They tested the performance of an instrument that used a continuous-wave pump-resonant singly resonant optical parametric oscillator (OPO) with known ethane concentration reference gas. The OPO was tuned to the vacuum wavenumber of 2,990.096 cm^–1^ to monitor ethane absorption. This demonstration resulted with a sensitivity of 300 ppt for ethane with the portability of the instrument as an added benefit. Later on, the authors developed a similar instrument [251] that used a CALOS setup with a CO overtone side-band laser which was tested for online ethane measurements of exhaled breath. The reported sensitivity of 500 ppt with an averaging time of 800 milliseconds was sufficient to record ethane in single exhalations. The only significant shortcoming was that the CO laser was too large for developing a portable instrument.

The requirement for a smaller laser source that allowed for portability was met by using an optical parametric oscillator that utilizes a pump-resonant singly resonant oscillator with two independent cavities [253]. The OPO has more power output in the mid-infrared than the difference frequency generation device used in the previous study. In addition, the frequency tunablility of the OPO allowed the selection of a spectral region for ethane monitoring where there are no significant interferences. The laser sources coupled with a CALOS setup resulted in impressive 6 ppt sensitivity for ethane detection. Their instrument was also able to measure methane and water in human breath.

There have been other studies that used different light sources or spectroscopic schemes that did not achieve the level of sensitivity of the above study. One study by Halmer *et al.* [254] used two laser sources (a widely tunable external-cavity diode laser and a diode-pumped monolithic Nd:YAG laser) for a difference-frequency mixing in periodically poled LiNbO_3_. The CALOS setup was employed to take online measurements of ethane and CO_2_ in exhaled human breath. The ethane measurement was taken in one minute, with a detection limit of 270 ppt. Another recent study uses a distributed feedback interband [255] cascade laser with an off-axis integrated-cavity output spectroscopic setup. The instrument had a 0.12 ppb detection minimum and was able to measure online ethane concentration in exhaled human breath.

A study was carried out to compare the CRDS technique with the traditional method of ethane measurement, gas chromatography-flame ionization (GC-FI), to show that CRDS techniques are viable and perhaps better than standard methods for breath analysis [256]. The comparison was made using the CALOS setup of Halmer *et al.* [254]. Several measurements were taken using both methods over a long period of time (one year for CALOS) and both intraday and interday reproducibility was investigated for a sample containing 5 and 50 ppb ethane. The results show that the CALOS technique yielded good agreement with that of GC-FI measurements but at a much faster (under one minute) processing time.

There have been several other reports that show the potential of high-finesse optical cavity-based spectroscopic techniques for the measurement of trace gases in human breath. However, these are mostly proof-of-concept studies or attempts to develop a portable instrument. Ammonia has been studied using pulsed QC laser in a CRDS setup [244] and also with cavity-enhanced optical frequency comb spectroscopy [244]. After their initial success designing a pulsed CRDS instrument [257] with 0.49 ppmv sensitivity for breath acetone detection, Wang *et al.* have reported an exploratory study of the performance of this portable breath acetone analyzer with human subjects [258]. Other biomarker molecules studied include carbon disulfide (CS_2_) [259] and very recently hydrogen cyanide (HCN) [260].

### 3.4. Future Outlook

The development of CRDS-based techniques is remarkable. Modifications and improvements on the already existing techniques continue to be reported. New approaches are being discovered. Many regions of the spectrum are probed using a multitude of laser sources. The commercial availability of CRDS-based instruments for several applications will surely increase its use in future studies. The use of supercontinuum radiation sources is a recent advancement of the techniques that allows a single light source to access a wide range of the spectral window [180-183]. The use of uncoated reflecting optics (prisms for example) is an advancement of the technique that allows for the use of cavities over a very broad wavelength range. The availability of room temperature QCL and DFB diode lasers will continue to improve the technique's portability. A recently demonstrated variant of the technique known as cavity-enhanced direct-frequency comb spectroscopy also allows for multiple species to be detected. The future of the CRDS technique will be in developing portable instruments that are able to detect multiple species (more than three) over a very wide spectral region.

## 4. Photoluminescence Sensing

### 4.1. Introduction

The use of coordination complexes for sensing applications of VOCs is currently attracting a great deal of attention as an alternative approach to polymeric materials. There are numerous current reports on chemical sensing that employ photoluminescence (PL) techniques. Hence, an overview is timely in order to follow the recent progress in the area of VOC sensor development. The review does not present every possible reference related to VOC sensing. Rather priority has been given to recent studies that place emphasis on solid state complexes and the molecular interactions with VOCs that permit analyte recognition through changes in PL properties. Since the PL process has now become a routine spectroscopic technique, no effort is made in this review to describe the technical aspect. Moreover, optical techniques and instrumentation will not be specifically emphasized. For more insight on optical transduction techniques pertinent to chemical sensing, the reader is directed to a recent review by MacCraith and co-workers [261]. Likewise, basic understanding of PL instrumentation can be gleaned easily from the abundant literature accounts and textbooks [262,263].

In addition to detecting VOCs, spectroscopic changes have also been used to identify other gases [264,265]. The conventional technique for detecting organic vapors and other gases employs the phenomenon of vapoluminescence. Vapoluminescence usually occurs as a change in emission intensity and/or shift in emission wavelengths, or as a change in infrared (IR) signature after exposure to VOC vapors [266-271]. A shift in PL spectra or intensity upon exposure to liquid solvents is termed as “solvoluminescence” [272], whereas a shift in emission or absorption wavelength after vapor exposure is termed as “vapochromism.” Ordinarily, PL sensors generate variable emission intensities in response to changes in analyte concentration under a sustained source of photoexcitation [273]. Undoubtedly, one crucial aspect with PL sensors is the efficiency of the luminescence collection [274].

Along with applications in environmental monitoring, chemical sensors can also be used as artificial noses, and serve purposes in the chemical and food industries [275,276]. In the last decade, the use of transition metal complexes as optical sensors for VOC detections has increased exponentially. Many works have bridged the preliminary findings discovered in the latter part of the 20^th^ century, to the works published during the first decade of the 21^st^ century. The review by Keefe *et al.* discusses the earlier developments (1994 to early 1999) of luminescence-based chemical sensing as they relate to the coordination chemistry of metals [277]. In the first part of this section, recent investigations of transition metal complexes that are used and/or have the potential for PL sensing are covered. This initial section includes transition metal complexes comprising of gold, mixed gold-silver, mixed gold-thallium, copper, and platinum-based systems, which have all proven to exhibit intriguing vapoluminescent properties. Progress in other metal complexes such as ruthenium, iridium, nickel, tin, and cobalt are also discussed to a lesser extent. Additionally, reports on zinc complexes are discussed separately since they have been studied extensively for host-guest systems. The subsequent sections cover complexes exhibiting on/off PL switching properties and other host-guest systems. Overall, the review will cover literature reported since the year 2000, with a few exceptions of earlier exemplary works.

### 4.2. Transition Metal Complexes for VOC Sensing

#### 4.2.1. Gold(I) Complexes

The photophysical properties of closed-shell, d^10^, transition metal systems have gained considerable attention in recent years. Gold(I) complexes are ordinarily colorless and non-emissive [278,279]. However, they can exhibit luminescent behavior due to self association through the formation of short Au⋯Au contacts known as aurophilic interactions [280,281]. Such contacts are dominant in crystalline solids but may also occur in solutions. These aurophilic interactions are unique because Au(I) complexes involve closed shell metal centers with no apparent valence electrons to connect the Au(I) centers. Nevertheless, they still manifest the tendency to self-associate through weak interactions in the ground state that become stronger in the excited state. Relativistic effects have also been noted to influence the self-association Au⋯Au interactions, as discussed recently by Pyykkö [282,283]. The high speeds of all the electrons as they move near a heavy nucleus leads to a radial contraction of the s and p orbitals resulting in an energetic stabilization [283]. Consequently, these weak interactions are revealed via apparent changes in their PL behavior. In the frozen state, aggregate formation has been reported to increase Au⋯Au interactions, causing enhancement in the PL intensity [284,285]. The review reported by Balch in 2007 [286], covers an adequate range of topics relevant to the luminescent properties of Au(I) complexes in two-coordinate situations. Most of the Au(I) complexes have been found to luminesce as solids and in this review emphasis is placed on the solid state spectroscopy.

While the Au⋯Au interaction is important for examining the sensing potential of Au(I) complexes, the recognition of other factors is also essential for a complete understanding of their PL properties. One of these factors is the proper choice of bridging ligand. The majority of the ligands used to bridge the Au⋯Au contacts have been phosphines or P-donors [287,288], cyanides [278,289], alkynyls [290], as well as other carbon, nitrogen, and sulfur donor ligands.

**Figure 2.**
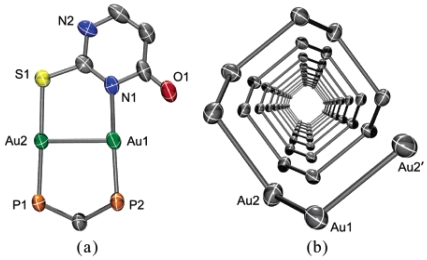
(a) View of cationic Au(I) thiouracilate containing dppm (phenyl rings omitted) and (b) helical arrangement of Au(I) ions with ligands omitted for clarity. Reproduced with permission from [291].

In 2003, Eisenberg and Lee [291] reported gold(I) thiouracilate complexes containing bis(diphenyl-phosphino)methane (dppm) that exhibited a phenomenon known as “tribochromism”. In contrast to triboluminescence, tribochromism is a sustained change in the emission spectrum upon the initial application of pressure [291]. The Au(I) complexes reported by Eisenberg are initially weakly emitting or nonemissive. After gentle crushing, however, the compound's emission is dramatically converted to an intense blue emission at 483 nm. It was concluded that the application of pressure to the complexes induces cleavage in the weakest links of the Au(I) helix (Figure 2b). Assefa *et al.* [289] also observed tribochromism in the complex [(TPA)_2_Au][Au(CN)_2_], where TPA = (1,3,5-triaza-7-phospha-adamantane), and concluded that since powdered Au(I)-chain complexes have increased surface areas, more sites of chain termination are exposed. Therefore, surface modifications may also induce emission changes through bonding rearrangements, and through formation of localized lattice defect centers [289].

A frequently cited work in the literature is the study by Eisenberg and co-workers [292]. This investigation has served as a catalyst for most of the works conducted over the last decade employing Au(I) complexes for VOC sensing applications. The Eisenberg group reported a “switching on” of orange luminescence upon selected VOCs interactions with a dimeric Au(I) dithiocarbamate complex [Au(S_2_CN(C_5_H_11_)_2_)]_2_. When the complex is exposed to vapors of aprotic solvents such as acetone, acetonitrile, dichloromethane, and chloroform, it exhibits structural changes where linear chains of Au atoms are formed with short intermolecular distances of 2.9617(7) Å. As a result of exposure to the VOCs, the complex becomes emissive. In the absence of these VOCs, the emission of the complex is completely quenched.

On the other hand, exposing the complex to vapors of protic solvents e.g., methanol or ethanol, provides a colorless and non-emissive crystal with a structural modification where the shortest intermolecular Au⋯Au distance is 8.135 Å. Thin films of the gold complex that were saturated in dichloromethane, exhibited an emission band at 630 nm. The absorption and emission maxima of the films exhibit little variation with the different aprotic solvents, except that when heated and dried the orange films become pale yellow and non-emissive. After the gold complex was diluted and glassed with DMF:MeOH:CH_2_Cl_2_, a broad emission at 563 nm was observed, which was significantly higher in energy than the thin films. Consequently, the longer chains of Au-complexes in the solid-state absorb and emit at longer wavelengths than the shorter chains in the glass since the Au⋯Au interaction is often restricted in glassed complexes.

**Figure 4.**
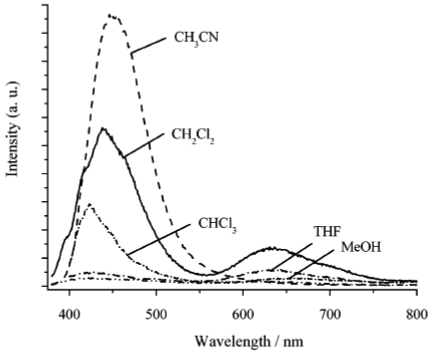
Emission spectra of {[(8-QNS)_2_Au(AuPPh_3_)_2_]}_2_·(BF_4_)_2_ measured in various solvents at 298 K. Complex concentration = 9 × 10^–5^ M. Excitation is at 320 nm. Reproduced with permission from [293].

The effect of solvent polarity on Au⋯Au interactions and the PL properties of Au(I) complexes were reported by Tzeng and co-workers [293]. The Tzeng group studied the hexanuclear complex, {[(8-QNS)_2_Au(AuPPh_3_)_2_]}_2_·(BF_4_)_2_ (8-QNS = quinoline-8-thiolate), which is portrayed in Figure 4. At room temperature, the solid state complex yields a single emission band at 587 nm. However, in various organic solvents intense higher-energy bands are exhibited at ∼420–480 nm, as well as lower-energy bands that show solvent-dependent features in the ∼620–680 nm spectral region. As shown in Figure 4, the polar solvents methanol, THF, and chloroform induce significant quenching of the low-energy bands in comparison to dichloromethane. In contrast, acetonitrile completely quenches the lower-energy band, while yielding the most intense higher-energy band at 477 nm. These solvent-dependent behaviors are hypothesized to arise from scrambling of the [PPh_3_Au]^+^ units within the complex [293].

A similar solvent-dependency has been demonstrated by Yam and co-workers [290]. It was observed that when tetranuclear Au(I) calixcrown alkynyls are excited by 350 nm, they yield emission bands at ∼587 nm when in solution with chloroform and as frozen matrices (glass). However, in the solid state, excitation at 370 nm yields lower energy bands that are red-shifted to ∼620 nm. The respective red-shift is clearly attributed to the presence of intramolecular Au⋯Au interactions which give rise to a narrowing of the HOMO-LUMO energy gap, most likely due to orbital splittings perturbed by intraligand excited states.

#### 4.2.2. Mixed Gold-Silver Complexes

Bimetallic systems involving mixed-metal interactions have also been explored recently. The “argento-aurophilic” Ag(I)–Au(I) interactions have been reported in the literature for potential VOC sensing applications [275,294-304]. For instance, recent works by Elosúa *et al.* have implemented Au-Ag complexes as optical fiber sensors [275,299,305]. Since both gold and silver have strong affinities for soft donor ligands, chelating phosphines have been used extensively to bridge two Au and/or Ag metal ions in close proximity [306]. Recent theoretical work by Fernández *et al.* describes how mixed-metal interactions enhance the dispersion forces by introducing dipolar interactions between dissimilar metals [307]. Consequently, this results in shorter metal-metal separations as compared with their respective homometallic analogues. The Fernández group [296] also characterized the structure of a [Ag(py)_3_][Au(C_6_F_5_)_2_] complex, which showed an extended ligand-unsupported chain of alternating gold and silver atoms. The short Au⋯Ag interactions are postulated to arise both from an attractive ionic(dipolar) contribution and from dispersion-type correlation effects [296]. Catalano and Horner [295] have also verified experimentally that shorter metal-metal separations exist in the mixed-metal complex [AuAg(dpim)_3_]^2+^ (dpim = 2-(diphenylphosphino)-1-methylimidazole).

**Figure 5.**
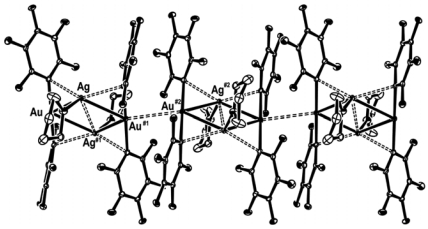
A portion of the polymeric structure of {Ag_2_(THF)_2_[Au(C_6_F_5_)_2_]_2_}_n_. Hydrogen atoms have been omitted for clarity. Reproduced with permission from [294].

The Fernández group [294] also examined the properties of the extended linear chain complex {Ag_2_L_2_[Au(C_6_F_5_)_2_]_2_}_n_ (L = Et_2_O, Me_2_CO, THF, or CH_3_CN). In this complex, pentafluorophenyl groups serve as asymmetrical bridges between the Au and Ag atoms as illustrated in Figure 5. Indeed, organic vapor inclusion into solid state complexes such as those containing Au–Ag linear chains can improve the packing within the crystal lattice by filling the voids with VOC molecules. A reversible solvent exchange often takes place when anisotropic packing adheres in a host material since vapor can be easily included into the unoccupied space in the lattice [294].

Catalano and Etogo used N-heterocyclic carbene (NHC) ligands to support closed-shell metal ion interactions [308]. Additionally, NHC ligands have been used as supports for weak Ag–Ag interactions [309-312]. In their 2005 report, Catalano and Etogo [308] observed a lack of correlation between emission energies and Au–Ag separations or the donor ability of a nitrile ligand in three nitrile-containing polymeric compounds. The origin of the emission energy shift was postulated to arise from the geometry of the metal chain and ancillary ligand electronic/steric properties rather than the metal-metal separation.

Ag(I) complexes are by far the most understudied compounds in regard to their PL behavior, despite being in the same family as Au(I) and Cu(I). A few accounts of their PL properties have been reported [313]. Omary and Rewashdeh-Omary have reports on PL (AgCN)_2_^−^ complexes [314-317]. However, very few investigations have been reported that discuss the interactions between Ag(I) complexes and VOCs, and therefore remains a wide-open area for further exploration.

#### 4.2.3. Mixed Gold-Thallium Complexes

Fernández and co-workers have reported potential sensor applications on numerous gold-thallium complexes that depend on bimetallic interactions [307,318-325]. A polymeric {Tl[Au(C_6_Cl_5_)_2_]}_n_ complex reported by Fernández *et al.* reacts with VOCs to yield products where the VOCs are coordinated to the Tl(I) centers [321]. The emission from this complex shifts to higher energies with increasing temperature due to increases in the metal-metal separations. The thermal expansion that results in increased metal-metal separation usually corresponds to an increase in the HOMO-LUMO gap energy [326].

**Figure 6.**
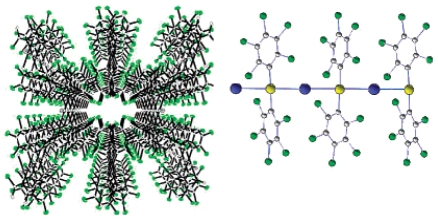
Crystal structure of {Tl[Au(C_6_Cl_5_)_2_]}_n_ viewed down the crystallographic *c*-axis. Inset: the polymeric molecular structure of {Tl[Au(C_6_Cl_5_)_2_]}_n_. Reproduced with permission from [327].

**Figure 7.**
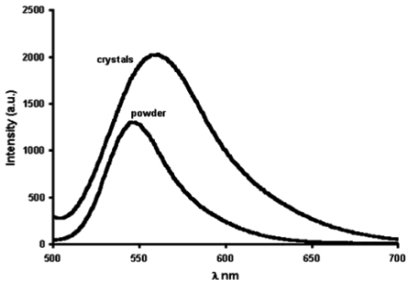
Comparison between emission spectra of crystals and powder of {Tl[Au(C_6_Cl_5_)_2_]}_n_ at 77 K. Reproduced with permission from [327].

The Fackler group also reported similar studies in 2003 [327], where a reversible vapochromic behavior is observed with the same {Tl[Au(C_6_Cl_5_)_2_]}_n_ complex when exposed to acetone, acetonitrile, triethylamine, acetylacetone, and other VOCs. At low temperatures, the emission of this complex exhibits red-shifts when exposed to all of the listed VOCs. This shift to lower-energy is attributed to thermal contraction of the Au-Tl distances that decreases the HOMO-LUMO gap. Furthermore, Figure 6 shows the {Tl[Au(C_6_Cl_5_)_2_]}_n_ crystal possessing channels running parallel to the crystallographic *c*-axis. The channels have diameters as large as 10.471 Å, which can easily accommodate vapor molecules into the lattice. A blue-shift occurs as the crystals are crushed to a powder which is shown in Figure 7. This phenomenon is attributed to the reduced spatial confinement of the electrons, which increases the oscillator strength between orbitals, thus demonstrating the quantum size effect.

Fernández and co-workers are also one of the first groups to synthesize mixed Au(I)-Cu(I) complexes. The photophysical properties of a [Cu{Au(C_6_F_5_)_2_}(N≡CCH_3_)(μ_2_-C_4_H_4_N_2_)]*_n_* (C_4_H_4_N_2_ = pyrimidine) complex were demonstrated for VOC sensing [302,328]. Since Cu(I) has a large affinity for nitrogen-donor ligands, more ligand variations and experimental studies are possible for mixed-metal systems.

#### 4.2.4. Copper(I) Complexes

**Figure 8.**
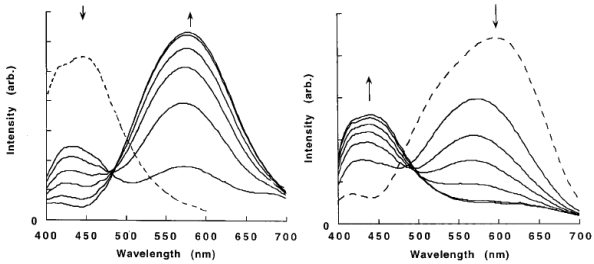
Emission spectral changes during the toluene induced [CuI(4-pic)]_∞_→[CuI(4-pic)]_4_ transformation (left), and during the pentane induced [CuI(4-pic)]_4_→[CuI(4-pic)]_∞_ transformation (right). Reproduced with permission from [266].

Copper(I) compounds, which previously did not receive much attention [329,330], are gaining interest for VOC sensing as well as being considered for use in host-guest systems [331]. Ford and co-workers [266,332] have reported earlier investigations of PL Cu(I) complexes, where vapoluminescence behavior was observed in two solid [CuI(4-pic)]_x_ complexes, one existing in a tetrameric form (x = 4) and the other in a polymeric form (x = ∞). Upon exposure to toluene vapor, the polymeric form exhibits a vapoluminescent shift in emission from 437 to 580 nm (Figure 8). In fact, toluene exposure results in a complex transformation from the polymeric form to the tetrameric form. However, the process is reversed when exposed to *n*-pentane vapors for three hours. Although the low cost of Cu(I) compounds makes them advantageous in practical sensing applications, the slow response times and dependability on particle dimensions compromises their suitability. For instance, the initial complex transformation is faster when it is finely powdered. For further detailed studies on Cu(I) complexes, the Ford group completed a thorough review outlining their PL properties [333].

More recently, Omary and co-workers reported on the photophysical properties of a trinuclear Cu(I) complex {[3,5-(CF_3_)_2_Pz]Cu}_3_, referred to as **Cu_3_** [334]. The emission behavior of the complex is affected by several factors including temperature, and exposure to benzene, toluene, and acetonitrile solvents. In addition, the Omary group introduced the concept of “concentration luminochromism”, a phenomenon that takes place when multiple visible emissions can be tuned by controlling the concentration of the **Cu_3_** complex.

#### 4.2.5. Platinum(II) Complexes

Platinum (Pt) complexes have been one of the most studied transition-metal complexes since the 19^th^ century and even well before the advent of coordination chemistry [335]. Ultimately, the ability of Pt(II) systems to selectively interact with specific compounds via vapoluminescence has served as a motivation for several studies [273,336-346]. Che and co-workers prepared four trinuclear [(CˆNˆN)_3_Pt_3_(μ_3_-L)]^3+^ complexes by treating them with phosphine ligands [347]. They reported that the absorption and emission energies of oligmeric cyclometalated Pt(II) complexes can be tuned to a large extent by the proper choice of tethering phosphine ligands. These Pt complexes are shown to serve as amplifiers for subtle concentrations of VOCs.

**Figure 9.**
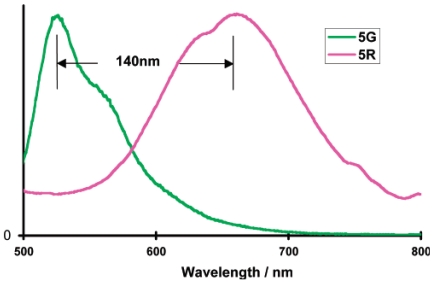
Emission spectra of **5-R** and **5-G** at 298 K in the solid state (4% in KBr). Reproduced with permission from [338].

In contrast to being subtle, Pt(II) complexes exhibit some of the largest vapochromic shifts ever seen among transition metal complexes. Du and co-workers [338] reported on a [Pt(TPPPB)Cl]Cl (TPPPB = 1-terpyridyl-2,3,4,5,6-pentaphenylbenzene) complex as showing high selectivity for VOCs such as methylene chloride, ethanol, ethyl acetate, and acetonitrile. Upon exposure to methylene chloride vapors, the emission of the [Pt(TPPPB)Cl]Cl complex exhibits blue-shifts from 654 to 514 nm. The resulting solid state PL spectra are shown in Figure 9. In the figure, the [Pt(TPPPB)Cl]Cl complex is referred to as **5-R** in the absence of VOC exposure, and **5-G** in the presence of the VOC vapor. The **5-R** complex possesses Pt⋯Pt distances of 3.30 and 3.34 Å, whereas the **5-G** complex contains significant Pt⋯Pt interactions with distances of 3.9092(9) and 4.5483(11) Å.

**Figure 10.**
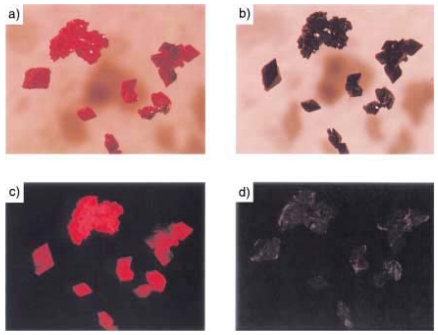
Photographic images of crystals of the syn isomer, illustrating vapochromic effects: (a) the light-red (desolvated) form in air and, (b) the dark-red form after exposure of (a) to acetonitrile vapor. Luminescence images of: (c) the light-red, and (d) dark-red forms. Reproduced with permission from [348].

In 2002, Kato *et al.* reported on a dinuclear platinum(II) complex that exhibited vapochromic changes in the presence of acetonitrile and ethanol [348]. Two geometrical isomers containing [Pt_2_(2,2′-bypyridine)(pyridine-2-thiolate)] ions were synthesized, one being a syn isomer in the form of dark-red crystals, and the other being an anti isomer in the form of orange needlelike crystals. The Pt⋯Pt separations for the syn and anti isomers are 2.923(1) and 2.997(1) Å, respectively. The intriguing observation was that the syn isomer crystals, which are initially dark-red, when left standing in air at room temperature become light red and exhibit red luminescence at 644 nm with a lifetime of 170 ns. Figure 10 shows images of the crystals after exposure to acetonitrile or ethanol vapor in which they become dark-red again with a shift in emission towards the infrared region at 766 nm.

The vapor-induced luminescence switching is evident in the syn isomer crystals; however, the sensitivity is reduced with increasing size of the organic vapor molecule. For instance, the *syn* isomer is less sensitive to isopropanol and exhibits no luminescence at all when exposed to *tert*-butanol despite both of them having similar vapor pressures to ethanol [348]. The source of vapochromism is attributed to the crystal structure of the syn isomer, where a co-planar-arrangement reveals a channel within its lattice that can hold one acetonitrile molecule per complex. In fact, X-ray analysis indicates several peaks of electron density within the channel, confirming that there are no distinct interactions between the main body of the complex and the acetonitrile molecule. The channel simply provides a pathway for organic vapors to easily penetrate the crystal. As a result, vapoluminescence occurs as Pt⋯Pt interactions are induced by the channels filling up with specific organic vapor molecules. The orange anti isomer crystal does not contain channels within its lattice, hence providing evidence for its lack of vapoluminescence.

**Figure 11.**
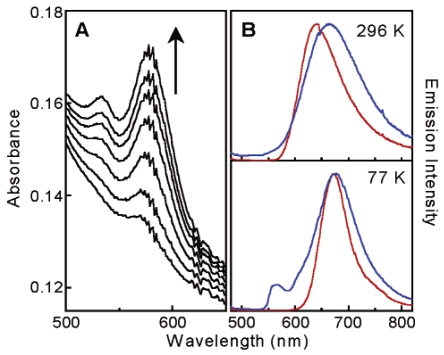
(a) UV-visible absorption spectra of a thin film of Pt(Me_2_bzimpy)Cl^+^ (PF_6_^−^ salt) recorded during exposure to acetonitrile vapor. (b) Solid-state emission spectra of Pt(Me_2_bzimpy)Cl^+^ (Cl^−^ salt) before (blue lines) and after (red lines) exposure to methanol vapor. Reproduced with permission from [349].

Since the early discovery of the Magnus' salt, [Pt(NH_3_)_4_][PtCl_4_], in 1828, optical investigations of Pt(II) salts continue to attract attention [267, 336, 343]. Connick and co-workers reported the vapoluminescent behavior of Pt(II) salts [349]. Cl^−^ and PF_6_^−^ anions of a Pt(Me_2_bzimpy)Cl^+^ (Me_2_bzimpy = 2,6-bis(*N*-methylbenzimidazol-2-yl)pyridine) complex were studied. PL studies conducted on the Cl^−^ salt in glassy solutions reveal emission bands at 545, 590, and 680 nm whose intensity depends on the complex concentration. The 680 nm band gains considerable intensity with increasing complex concentration, indicating possible formation of emissive aggregates. At room temperature, the Cl^−^ salt in the solid state exhibits a broad band near 670 nm that blue-shifts and becomes sharper upon exposure to methanol vapor (Figure 11b).

When the Cl^−^ salt is cooled, the emission bands sharpen further and red-shift to 685 nm both before and after exposure to methanol, indicating that thermal effects cause lattice contractions which shorten the Pt⋯Pt interactions. The solid-state emission studies of the Cl^−^ salt suggest that sorption of methanol vapor increases the Pt⋯Pt interactions, which possibly changes the orbital character of the lowest emissive state. Similar behavior is observed for the PF_6_^−^ salt when exposed to acetonitrile vapor except emission maxima are more red-shifted due to stronger Pt⋯Pt interactions.

More recently, Connick and co-workers have also demonstrated how vapochromic salts of Pt(II) systems may present advantages over other vapochromic Pt(II) systems in terms of selectivity, color change, and response speed. Since these characteristics are drastically dependent on the counteranion, this provides a strategy for tuning the response of the complex [350]. At the molecular level, anions and VOC molecules fill the voids between the columns of cations in vapochromic Pt(II) salts. Similarly, VOC molecules line channels along the *c*-axis, also providing a possible route for diffusion of vapors in and out of the host lattice.

Pt(II) coordination compounds are also among the most prevalent form of extended linear chain (ELC) compounds used in VOC sensing [351]. Coordination complexes with Pt(II) centers are often substituted by palladium centers due to their similar chemical properties. However, the two atoms show considerable differences in the manner of their ELC structures [352] and crystal packing [353]. As a result, the capacity for Pt and Pd complexes to accommodate VOCs through lattice channels can vary. The lack of reversibility is a major impediment in some of the Pt(II) ELC complexes that may have the potential for VOC sensing. Drew and co-workers [351] investigated a Pt^II^(CN-*i*-C_3_H_7_)_2_(CN)_2_ complex that shows a considerable spectroscopic change upon exposure to benzene. The selectivity of this crystalline complex for benzene exhibits remarkable shifts from a yellow emission at 558 nm to a blue emission at 484 nm upon the formation of a benzene solvate. However, the removal of the benzene from the resulting solvate yields a 50% reduction in both the absorptivity and the emission intensity. Furthermore, a repeated exposure of the same sample to benzene does not match the initial emission spectrum of the benzene solvate. The loss of reversibility is attributed to the molecular structure of the Pt^II^(CN-*i*-C_3_H_7_)_2_(CN)_2_ complex because it lacks the necessary molecular channels to accommodate the penetration of benzene into the lattice. Consequently, the exposure of benzene forces individual crystallites to open, destroying the long-range order of the lattice. This renders crystalline degradation that can only be reversed by exposure to an acetonitrile precursor. Indeed, previous studies have shown that the choice of precursor molecules can also effect Pt⋯Pt interactions since they can vary the size and structures of nanoscale aggregates [344].

#### 4.2.6. Other Metal Complexes

**Figure 12.**
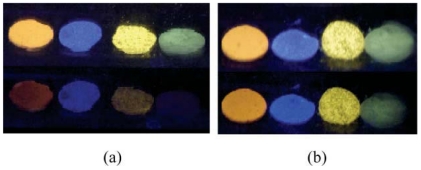
Visible detail of the initial versus diminished light emission seen from the array of Sn^2+^ pressed pellets (left to right: SnSO_4_, SnPO_3_F, Sn(CH_3_SO_3_)_2_, Sn(CF_3_SO_3_)_2_) when exposed to (a) 100 ppm of pyridine and (b) 1 ppm pyridine. Reproduced with permission from [354].

Other metal complexes such as tin(II) salts exhibit remarkable PL properties that are often quenched upon exposure to organic vapors. Baldauff and Buriak reported an optical sensing array comprised of four luminescent Sn(II) salts that are selectively quenched by amine vapor [354], where the quenching events vary in terms of their reversibility. The study consisted of the sulfate, methanesulfonate, triflate, and fluorophosphate salts of Sn(II), which are shown in Figure 12. The quenched blue emission from the fluorophosphate salt is consistently reproducible after simple N_2_ purging. In contrast, the sulfate and methanesulfonate salts are only partially regenerated after exposure to trifluoroacetic acid (TFA) vapors, while the triflate salt yields no reversibility after N_2_ purging or TFA exposure. Hence, Baldauff and Buriak have opened the door for more investigations to utilize Sn(II) compounds for optical chemical sensing.

Navale and Mulla [355] reported on the use of Sn as a dopant in ZnO crystals for vapor sensing. The inclusion of Sn into the ZnO crystals induces a significant morphological change that causes a reduction of sensitivity towards acetone vapors. Also as a result of the doping, the emission band shifts dramatically from the UV region to a green emission. Gu and co-workers have also reported potential sensing applications of luminescent SnO_2_ compounds in the form of thin films [356] and nanoparticles [357,358].

Ruthenium(II) complexes have also been considered for sensing polar organic solvents [359]. McGee *et al.* has reported a ruthenium [Ru(5,6-Me_2_Phen)_3_]tfpb_2_ (5,6-Me_2_Phen) = 5,6-dimethyl-1,10-phenanthroline; tfpb^−^ = tetrakis(bis-3,5-trifluoromethylphenylborate) complex that was examined for the sensing of benzene and oxygen [265]. The solid Ru(II) complex demonstrates a very rapid and reversible vapochromic shift of emission from 572 to 558 nm when exposed to benzene alone, while exposure to oxygen alone results in PL quenching. In contrast, when exposed to benzene and oxygen simultaneously, the Ru(II) crystals uptake benzene molecules preferentially and the diffusion of oxygen into the lattice is restricted. Therefore, the emission intensity does not exhibit significant quenching. Nevertheless, upon the removal of benzene from the system, the oxygen-induced quenching is restored. The efforts of the McGee group are ongoing in an attempt to meld these two sensor mechanisms into a single functioning system. Coincidentally, some luminescent Ru(II) complexes have also been reported as being highly sensitive in the presence of moisture [360].

**Figure 13.**
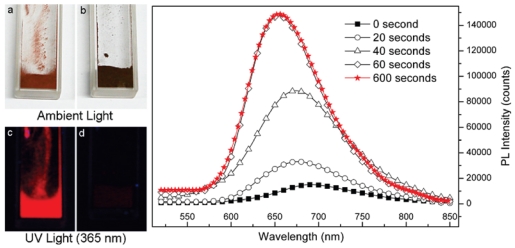
Photographic images of (a) the red form, and (b) the black form of PIr(qnx). Luminescence images of (c) the red form, and (d) the black form of PIr(qnx). Photoluminescence spectra of the black form exposed to acetonitrile vapor at different periods of time. Reproduced with permission from [361].

Luminescent iridium complexes have also been studied for their unique selectivity of VOCs. Liu *et al.* [361] reported a vapoluminescent cyclometalated heteroleptic iridium complex [iridium(III)-bis(2-phenylpyridinato-N,C2)(quinoxaline-2-carboxylate); PIr(qnx)] that was synthesized into two forms (black and red forms). The black form is weakly luminescent at 692 nm, with a short lifetime of 43 ns. The black form also transforms into the red form when exposed to acetonitrile or propiononitrile vapor, but exhibits no response when exposed to 15 other VOCs. The red form displays intense PL at 654 nm with a lifetime of 130 ns, demonstrating selectivity for acetonitrile vapor. Photographic images and solid state PL spectra are shown in Figure 13. The acetonitrile selectivity of the black form is attributed to the weak interaction of the left-over solvent molecules within the lattice. Since the black crystals were grown from ethanol/chloroform, the oxygen atoms of the trapped ethanol within the lattice are held at a distance of 3.27 Å from the hydrogen atoms of the PIr(qnx) complex, allowing ethanol molecules to be easily substituted by acetonitrile.

Reports on the use of cobalt for VOC sensing applications are limited. Beauvais *et al.* [362] reported on clusters of [Re_6_Q_8_(CN)_6_]^4-^ (Q = S, Se) which were used to space out hydrated Co(II) ions creating porous materials that display dramatic color changes upon exposure to certain organic solvents. Complexes of [Re_6_Q_8_(CN)_6_]^4-^ anion with various Co(II) ions such as [Co_2_(H_2_O)_4_]^4+^, have flexible structural frameworks that can expand to accommodate the incoming solvent molecules.

Similar vapochromic shifts in absorption spectra were also observed for some nickel(II) complexes. Baho and Zagarian [363] have reported a series of Ni(II) complexes coordinated with a diphenyl-(dipyrazolylmethane) (dpdpm) ligand. Upon exposure to acetonitrile vapor, the solid state complexes exhibit vapochromic shifts from green to light blue in approximately eight minutes. The shift is attributed to a reversible coordination of acetonitrile at the Ni center. Thermochromic behavior is also observed in these Ni(II) complexes upon exposure to higher temperatures [363].

### 4.3. On/Off Photoluminescence Switching

Molecular sensors based on PL switching are usually governed by weak noncovalent interactions. The on/off PL switching phenomenon employs versatile qualities that allow rapid interaction between sensing compounds and various analytes. An example of on/off PL switching was reported by Wong and co-workers [273], where a series of six different Pt(II) complexes containing functionalized arylacetylide ligands were studied. The on/off PL switching behavior for the compound [(*t*Bu_2_bpy)Pt(C≡CAr)_2_] (*t*Bu_2_bpy = 4,4′-bis-*tert*-butyl-2,2′-bipyridine, Ar = 4-pyridyl (complex **1**) was studied as a thin film by exposing it to selected vapors of dichloromethane and chloroform. Upon exposure to both vapors, a “switching on” of intense emission takes place at 520 nm. When the vapors are removed, the PL is “switched off”, and only a very weak emission is observed at ∼527 nm. The “switching on” of the PL is attributed mainly to the presence of molecular channels within the lattice of the complex that harbor sorption of VOC molecules. The Wong group suggested that hydrogen bonding, intraligand, and other noncovalent interactions also play significant roles in the on/off PL switching phenomena.

**Figure 14.**
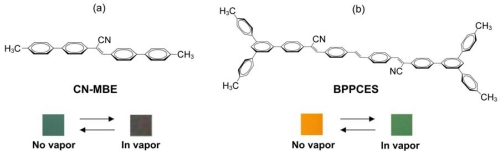
Chemical structures of: a) CN-MBE, and b) BPPCES. The photos show PL color changes of CN-MBE and BPPCES without vapor (left) and in vapor (dichloromethane) (right), respectively. Reproduced with permission from [364].

In addition to transition metal-based systems, the employment of totally organic molecular systems has also been utilized in “on/off” PL switching. Park and co-workers reported “on/off” and dual PL switching in a new class of organic molecules [364]. The Park group explored a molecular phenomenon known as aggregation-induced enhanced emission (AIEE) that occurs in the solid state. Two different compounds, 1-cyano-trans-1,2-bis-(4′-methylbiphenyl)ethylene (**CN-MBE**) and 4,4′-bis-((2-((4-(3,5-bitolyl)phenyl)phenyl)-2-cyano)-transethenyl)-trans-stilbene (**BPPCES**) exhibit emissions at 488 and 550 nm, respectively. Upon exposure to dichloromethane, chloroform, and tetrahydrofuran (THF) vapors, the CN-MBE emission at 488 nm is “switched off,” and the BPPCES emission at 550 nm is switched to 512 nm. The chemical structures and PL switching are presented in Figure 14. The PL switching of these compounds is fast and reversible. This sensitivity is attributed to the compounds being readily soluble in these organic solvents. As a result, the vapors are more likely to isolate the AIEE molecules from aggregation, triggering the dramatic PL changes. In contrast, poor PL sensing is observed upon exposure to *n*-hexane, methanol, and water vapor due to poor solubility of the complexes in these solvents. An earlier study on CN-MBE and PL switching was also reported by the Park group, but with greater emphasis on the effect that particle size has on PL properties [365].

### 4.4. Host-guest Molecules

An essential constituent of supramolecular chemistry is the process where the inclusion of organic guest molecules initiates luminescence. The assembly of molecules accommodating other molecules is studied as a topic of chemistry termed as “host-guest” chemistry. The preparation of host molecules that selectively recognize specific VOCs through measurable changes in luminescence has been a hot topic of study [366-368]. In the current section, emphasis will be placed on the extent to which a vapor is sorbed by a host molecule and the dependence of the PL properties on the host-guest interaction.

In 2002, Buss and Mann [369] synthesized a vapoluminescent Pt(CN-p-(C_2_H_5_)C_6_H_4_)_2_(CN)_2_ host, which, upon recrystallization, yields an orange crystalline form and a purple amorphous form. It was observed that the orange form exhibits a blue-shifted emission upon the inclusion of toluene and mesitylene guest molecules. The higher crystallinity of the orange form makes it a more advantageous sensor due to its thermodynamic stability, and its unresponsivity to water and air. Furthermore, the higher packing efficiency allows incorporation of more guest solvent molecules. The orange complex is also highly soluble in common solvents, making it castable for film fabrication. The X-ray powder diffraction studies of the orange form suggest that the blue-shift in the emission spectrum upon exposure to toluene arises from increased Pt–Pt separation, and hence, an increased HOMO–LUMO gap.

In addition to Pt(II) complexes, Zn(II) compounds have also been studied for host-guest interactions [370]. Das and Bharadwaj reported a luminescent [Zn(bpy)(aba)_2_] {bpy = 2,2′-bipyridyl and aba = 4-dimethylaminobenzoate} complex that shows selectivity toward nitrobenzene in both solution and vapor phase [371]. Upon exposure to nitrobenzene, the complex demonstrates a dramatic color change from white to red accompanied by a thermally reversible quenching of luminescence. The Zn(II) complex initially exhibits a consistent broad emission at 520 nm when exposed to various organic guests such as benzene, toluene, and xylene. However, a drastic change in luminescence quantum yield is observed as the emission intensity decreases with the inclusion of a nitrobenzene guest.

**Figure 15.**
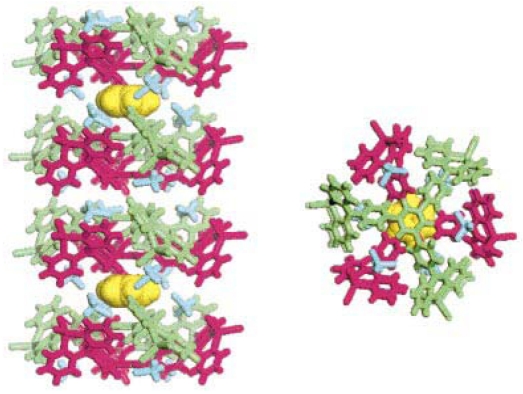
Stick/space-filling diagrams showing the sandwiched arrangement of benzene and [(ZnCl_2_)_3_(TPDPB)] in crystal A, viewed along *c*-axis (left) and viewed down the *c*-axis (right). Benzene: yellow, space filling; CH_2_Cl_2_: light blue, stick. The interlocked pairs of molecules of [(ZnCl_2_)_3_(TPDPB)] are shown as red and green, respectively. Reproduced with permission from [372].

Likewise, luminescence quenching in a Zn(II) complex was also reported by Wang and co-workers [372]. The Wang group coordinated 1,3,5-tris (*p*-(2,2′-dipyridylamidipyridylamino) phenyl)benzene (TPDPB) ligand to ZnCl_2_ to form a star-shaped blue emitting [(ZnCl_2_)_3_(TPDPB)] complex, which is shown in Figure 15. It was also observed that the solid-state PL intensity of the complex is partially quenched upon inclusion of a benzene guest. The quenching phenomenon is attributed to *π* − *π* stacking, face-to-face or edge-to-face interactions between benzene and the [(ZnCl_2_)_3_(TPDPB)] complex [372].

Chen and co-workers [373] reported a novel three dimensional metal-organic framework (MOF) constructed by Zn_4_O clusters coordinated with 1,4-benzenedicarboxylate (bdc) and 3,3′,5,5′-tetramethyl-4,4′-bipyrazolate (bpz) ligands. The complex is composed of hydrophobic channels that enable incoming organic solvent guests to move freely in and out of the molecular pores. Upon interaction with the organic guests benzene, toluene, and p-xylene, the emission bands are observed at blue-shifted positions of 435, 465, and 466 nm, respectively. The blue-shifts are attributed to the mechanistic inclusion of the rigid guest molecules, which weakens the skeletal vibration, stabilizing the MOF, and enhancing the intraligand excitation energy [373]. As a result, more intense higher energy emissions are observed.

### 4.5. Photoluminescent Porphyrins

**Figure 16.**
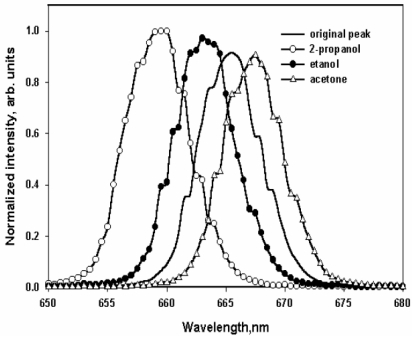
The emission spectra thin films of TiO_2_ nanoparticles coated with porphyrin ratio 1:2 at the original peak and in presence of 2-propanol, ethanol, and acetone. Reproduced with permission from [376].

Porphyrin-based molecules are favorable for VOC sensing since their optical properties can be tuned by incorporating different metals into their center rings [374,375]. Yusoff *et al.* reports the potential VOC sensing of thin-films of TiO_2_ nanoparticles coated with a porphyrin dye [376]. The volume ratios of porphyrin to TiO_2_ were 1:2, 1:3, 1:4 and 1:5. The emission spectrum exhibits blue-shifts with each increasing ratio of porphyrin volume. After exposing the thin films to ethanol, acetone, and 2-propanol, samples with porphyrin to TiO_2_ ratios of 1:2, 1:3, and 1:4 interact with the 2-proponol and ethanol and the emission characteristically blue-shifts, while interaction with acetone results in a red-shifting. It appears that the 1:5 ratio is non-emissive, while the 1:2 ratio exhibits the most sensitivity to the VOCs due to its smaller grain size. The increased surface area provides more opportunity for interaction with organic vapors. The resulting emission spectra for the 1:2 ratio are shown in Figure 16.

Porphyrins have also found use in the detection of some volatile organic acids. Yu and co-workers [377] have reported on the application of porphyrins for sensing of picric acid. Despite playing an important role in organic synthesis and drug analysis [378], picric acid is highly volatile and hazardous. When a porphyrin dimer is combined with anthracene, PL quenching is observed after exposure to various concentrations of picric acid. The Yu group demonstrated an efficient transfer of luminescence energy from the anthracene donor to the porphyrin acceptor that is highly influenced by a picric acid analyte. It was postulated that the porphyrin acceptor contains two porphyrin rings in one molecule which provides a supramolecular environment for the picric acid molecule.

**Figure 17.**
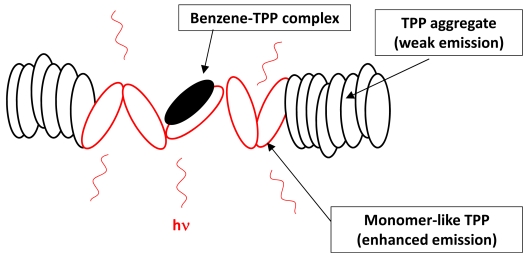
Illustration of Benzene-TPP complex formation in TPP aggregates. Modified version from [379].

One noteworthy invention was also reported by Levitsky and Krivoshlykov [379], where solid films of porphyrin aggregates were employed to detect vapors of benzene, alcohol, chloroform, and dimethyl methylphosphonate (DMMP). Ordinarily, the aggregation of porphyrin molecules leads to depression of emission due to the self-quenching effect. However, this invention utilizes the binding of analyte molecules to a porphyrin sensitive thin film layer to form porphyrin-analyte complexes. Subsequently, this complexation destroys the aggregate structure releasing the porphyrins into a monomer-like state where freely exposed porphyrins exhibit a strong luminescence enhancement. Initially, the porphyrin dye is in the aggregated form of a Zn-centered tetraphenylporphyrin (Zn-TPP). After interaction with benzene vapors, a benzene-TPP complex is formed that releases more TPP from aggregation. As a result, enhanced emission bands are observed at 650 and 710 nm, with a shoulder at ∼730 nm. The benzene analyte-binding process is illustrated in Figure 17.

### 4.6. Additional Remarks

This section of the review was intended to thoroughly discuss solid state PL complexes/materials, conceptually analyze their molecular interactions with VOCs, and evaluate their potential sensing capabilities. Ultimately, the planned field of reference was to stay within the confines of works reported in the last ten years. Despite the thorough nature of this review, only a limited number of topics relevant to PL-based sensing have been covered. Due to time and space limitations, the review of totally organic-based VOC sensing compounds such as luminescent β–cyclodextrins [380-393] was omitted. Also, recent reports on PL-based microarrays and artificial noses [394-400] were not discussed in detail. Moreover, the significant works on PL-based thin films [401-403], silica gels [404,405], and/or other nanomaterials [406-408] have not been targeted for this review. Likewise, novel studies such as those involving VOC interactions with PL marine diatoms were also excluded [409,410]. Certainly, PL-based sensing is a rapidly growing area that will remain wide-open for future innovative investigations and more frequent reviews.

## Figures and Tables

**Figure 3.**
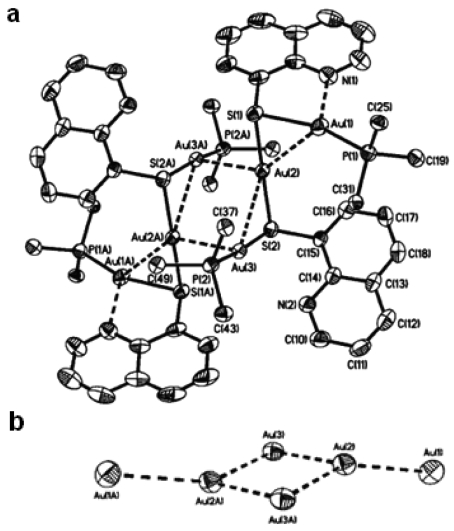
(a) Dimeric aggregate of complex {[(8-QNS)_2_Au(AuPPh_3_)_2_]}_2_·(BF_4_)_2_ cations where the hexanuclear gold(I) supermolecule is connected by intermolecular Au(2)⋯Au(3A) contact of 3.1135(3) Å. The coplanar arrangement of the six metal ions is shown in (b). Reproduced with permission from [293].

**Table 1.** Laser-based spectroscopic applications to environmental solids.

**Element**	**Sample**	**Method**	**LOD**	**Notes**	**Reference**
Tl	Sediment	LEAFS	0.5 ppb	HNO_3_-HF Dissolution	[75]
Al, Ba, Ca, Fe, Mg, Mn, Na, Si, Sr, Ti	Sediment	LIBS	Not given	Various sampling systems evaluated, samples were dried, instrument was ship-board	[76]
Al, Ba, C, Ca, Fe, K, Li, Mn, Na, Si, Sr, Ti	Sediment	LIBS	114-566 ppm	Underwater measurement using many techniques	[72]
Ba, C, Ca, Cu, Fe, O, S, Zn	Minerals	LIBS	Not given		[77]
C, Br, Cl	Organic solids	LIBS	0.011, 0.054	LODs refer to Br/C and Cl/C ratio, respectively	[78]
As, Cr, Cu	Treated wood waste	LIBS	Not given	X-ray fluorescence also evaluated	[79]
As, Ca, Cr, Cu, Na, Zn	Treated wood	LIBS	Not given	Wood discriminated based on treatment, compared to digestion and AAS	[80]
Many (21)	Ore	LIBS	2-16 ppm	Contaminant screening in open pit mines	[81,82]
P, Si	Phosphate ore	LIBS	Not given	Removal of high silica pebbles, single shot analysis	[83]
Al, Fe, K, Si, Mn, P	Iron ore	LIBS	Not given	Principle component regression, samples powdered, pressed	[84]
Many (34)	Beryl	LIBS	Not given	Differentiation of country of origin	[85]
Ba, Ca, Cd, Cr, Mg, Mn, S, Ti	Slag	LIBS	6-16 ppm	Risk assessment of steel plant waste	[86]
C	Soil	LIBS	300 ppm	Also examines near-IR spec. and inelastic neutron scattering	[87]
C, N	Soil	LIBS	Not given	Acid washing used to distinguish organic and inorganic C	[88]
Ba, Ca, Fe, K, Mg, Ni, P, S	Soil	LIBS	7-12 ppm	2% precision	[89]
Cr	Soil	LIBS	Not given	Principle component regression, samples pressed into pellets	[90]
Al, Ba, Ca, Cr, Fe, K, Mg, Na, Sr, Ti, V, Zr	Soil, contaminated	LIBS	2-12 ppm	Soil contaminated by coastal oil spill, compared to ICP- emission spectroscopy	[91]
Fe, Cr	Soil, rocks, vegetation	LIBS	2 ppth	Laboratory-based assessment on contamination of remote coastal areas from industrial activity	[92]
Al, B, Ba, C, Ca, Fe, H, Mg, Mn, Na, Si, Sr	Marble and wood	LIBS	Not given	Immersed in seawater, only marble yielded quantitative results	[73]
Fe, Mg, Si	Asbestos	LIBS	Not given	Discrimination between serpentine asbestos, amphibole asbestos, and cement	[93]

**Table 2.** Laser-based spectroscopic applications to environmental liquids.

**Element**	**Sample**	**Sample Preparation**	**Method**	**LOD**	**Notes**	**Reference**
Tl	Water	Direct analysis	LEAFS	0.03 ng/L		[96]
Pb	Water	Direct analysis	LA-LEAFS	35 ppb		[122]
Pb	Water	Pneumatically sprayed	LIBS, LA-LEAFS	75 and 4.3 ppm		[123]
Cr	Water	Preconcentration using cation exchange resin	LIBS	500 ng/L	Sample preparation allowed speciation of Cr^III^ and Cr^IV^	[124]
Al	Seawater	Autosampler	ETA-LEAFS	5 ng/L	CCD camera detector	[125]
Pb	Seawater	Autosampler	ETA-LEAFS	0.3 ng/L	CCD camera detector	[126]
Cr	Seawater	Direct analysis	LIBS	60 ppm		[127]
Ca, Mg, Na	Seawater	Direct analysis	LIBS	Not given	Sampled at the surface, in air at atmospheric pressure	[128]
Al, Ba, Cr, Cu, K, Mg, Na, Ni, Pb, Si, Ti, Zr	Wastewater	Evaporation of water	LIBS	1-301 ppm	Paint manufacturing plant effluent	[129]
Ca, Cu, Fe, K, Mg, Mo, Na, Ni, Zn	Crude oil	Serial distillation, heating in furnace to form pellet	LIBS	2-4 ppm	Compared to ICP-OES	[130]

**Table 3.** Laser-based spectroscopic applications to environmental gases and aerosols.

**Element**	**Sample**	**Sample Preparation**	**Method**	**LOD**	**Notes**	**Reference**
Hg	Air	Direct analysis	LEAFS	0.1 ng/m^3^	Double resonance excitation, quartz cell	[134]
Al, Ca, Cr, Cu, Mg, Mn, Na	Urban aerosol	PM2.5 cyclonic inlet and virtual impactor	LIBS	15-185 fg	Field experiment at Pittsburgh Aerosol Supersite	[135]
Al, Ba, Ca, Cl, Fe, Mg, Na, P	Urban bioaerosol	Direct analysis	LIBS	Not given	Comparison to aerosol mass spectrometry, 0.1–2 μm aerosols can be quantified	[133]
Many (14)	Aerosol	Cascade impactor, greased aluminum substrate	LIBS	Not given	Particles from steel making, composition shows size dependence	[136]
Al, C, Ca, Fe, N, Na, O, P, S, Si	Inorganic aerosol	Impactor or passive deposition	micro-Raman	Not given	>1 μm diameter, organic and mixed particles more problematic	[137]

**Table 4.** Isotope studies with CRDS.

**Species**	**Technique**	**Laser**	**Reference**
^13/12^C in CO_2_	CRDS	Near IR-External Cavity Diode Laser	[232]
^13/12^C in CO_2_	OA-CEAS	DF Diode Laser	[233]
^13/12^C in CO_2_	CRDS	Near IR Diode Laser	[234]
^13/12^C in CO_2_	OF-CEAS	DF Diode Laser	[235]
^13^C in CO_2_	NIR-CW-CRDS	DF Laser Diodes	[236]
D/H, ^18^O/^16^O	OA-ICOS	Not known	[237]
Water	WS-CRDS	Tunable Diode Laser	[238]
